# Actin conformation dynamics precede force generation in cytotoxic T lymphocytes

**DOI:** 10.1038/s42003-026-11011-3

**Published:** 2026-09-25

**Authors:** Adam M. Rochussen, Aybaran O. Kebabci, Alex K. Winkel, Kristian Franze, Daniel A. Fletcher, Gillian M. Griffiths, Anna H. Lippert

**Affiliations:** 1 Cambridge Institute for Medical Research, University of Cambridge, Cambridge, UK; 2 Institut für Systemimmunologie, Julius-Maximilians Universität Würzburg, Würzburg, Germany; 3 Department of Physiology, Development, and Neuroscience, University of Cambridge, Cambridge, UK; 4 Medical Institute of Biophysics, Friedrich-Alexander-Universität Erlangen-Nürnberg, Erlangen, Germany; 5 Max Planck Zentrum für Physik und Medizin, Erlangen, Germany; 6 Department of Bioengineering, University of California, Berkeley, CA USA; 7 Department of Immunomedicine, University of Regensburg, Regensburg, Germany; 8 Present Address: Salk Institute for Biological Studies, La Jolla, CA USA; 9 Present Address: Department of Cell Biology, Yale School of Medicine, New Haven, CT USA

**Keywords:** Imaging the immune system, Biopolymers in vivo

## Abstract

Cytotoxic T lymphocytes (CTLs) are cells of the adaptive immune system that are able to recognise and kill cancerous or virally infected target cells. The biochemical basis of CTL-mediated killing has been examined in great detail. Recent work has underscored the importance of physical forces in T cell function too, but how CTLs sense and exert force during migration and killing is not fully understood. Here, by expressing actin conformation probes based on the CH domain of utrophin, we directly visualize regions of altered F-actin conformation in primary CTLs during migration and killing. By combining these probes with traction force microscopy, we correlate external force with the internal cytoskeleton. We show that actin conformation is regulated upstream of force production, and that force exertion at the cytotoxic immune synapse temporally follows actin conformation dynamics. Our work offers insight into the relationship between cellular force production and F-actin conformation in important primary immune cells.

## Introduction

Cytotoxic T lymphocytes (CTLs) are important cells of the adaptive immune system responsible for the specific elimination of virally-infected and oncogenic cells presenting foreign peptides that can be recognised by the T-cell receptor (TCR). These are highly mechanosensitive cells, encountering a wide range of stiffnesses from the soft thymus to stiffer inflamed cells^1^. CTLs depend on force transduction in multiple different contexts: (i) migration, (ii) activation, and (iii) CTL-mediated killing^2^.

T cells exhibit durotaxis, that is, enhanced migration on and towards stiffer substrates^3^, likely mediated by actin-based adhesion modules, as has been shown in other cell types^4^. Since inflammation increases the stiffness of antigen-presenting cells (APCs)^5^, this durotaxis likely aids in the targeted migration of T cells during infection. Force-sensing and force-exertion go hand-in-hand for migratory cells, potentiating their migration through a range of tissues of differing stiffnesses^6,7^. For example, it is thought that the polymerisation of branched actin in leading-edge lamellipodia actively pushes cells forwards under certain modes of migration^8^.

After migration, activation of T cells is also highly stiffness dependent. T-cell activation, which occurs downstream of TCR ligation, is modulated by co-stimulatory receptors, cytokine signalling, and metabolic inputs^9^. Recently, the physical stiffness of the APC and the transduction of forces have also emerged as essential factors, with stiffer APCs leading to enhanced  T cell activation^5,10,11^. Even high concentrations of high-affinity TCR ligands cannot induce T-cell activation if the opposing surface is too soft^12^. Notably, this property of T-cell activation is one that is potentially exploited by cancerous cells, with softness correlating with metastatic potential and evasion of CTL-mediated killing^13–16^.

T cells not only sense force, but they also exert force, particularly at the immune synapse where forces are thought to play an important role in CTL-mediated killing^17–19^. Notably, much mechanobiology of the immune synapse has depended on tractable immortalised T-cell lines, such as Jurkat T cells, yet key differences between these model systems and primary T cells, as well as differences between CD4^+^ and CD8^+^ T cells, have been highlighted by others^11,20,21^.

The actin cytoskeleton underpins the ability of T cells to generate forces, with myosin motors and varied actin polymerisation machineries all playing a role^22,23^. F-actin is highly dynamic at the cytotoxic immune synapse. Initial contact and T-cell activation result in a characteristic ring of F-actin, with a depleted zone in the centre where TCR signalling, centrosome polarisation, and cytolytic protein secretion occurs^24–26^. Retrograde actin flow and actomyosin arcs work to concentrate transmembrane receptors into the centre of the synapse, sustaining the signalling environment^27^. Over time, the F-actin recovers across the synapse, blocking further secretion and marking the termination of the synapse before the cell detaches^28^.

How the actin cytoskeleton exerts and responds to force is a subject of continued interest. One hypothesis posits that filament twist, altered by compression/tension, can affect the binding of actin-binding proteins, and thus transduce a physical force into a biochemical signal^29^. Visualising different F-actin conformations in living cells is possible via genetically encoded actin-binding proteins fused with fluorescent proteins, with different probes binding to the cytoskeleton in varied ways^30^. For example, tandem calponin homology domains of the actin-binding protein utrophin (“utrn-CH”) are known to preferentially bind to F-actin near the uropod of migrating primary CD4^+^ T cells^31,32^, whilst the seventeen-residue polypeptide that constitutes LifeAct binds evenly across the length of migrating cells but fails to bind highly twisted cofilin-bound actin rods^33,34^. Structural analysis of utrn-CH revealed F-actin binding across three monomers, supporting its potential sensitivity to actin filament twist^35^. Previously, variants of utrn-CH with altered F-actin-binding properties were developed, including the Q33A, T36A, K121A mutant (“utrnLAM”), which is enriched in lamellipodial networks in living cells^36^. In a neutrophil-like cell line, simultaneous expression of the wild-type utrn-CH (“utrnWT”) and utrnLAM showed differential localisation along the front-rear axis during migration^36^. These utrn probes could be easily displaced by physiologically relevant concentrations of actin-binding proteins in vitro, implying they could be used as a ratiometric reporter of actin filament conformation within cells without impacting function, although this has not been tested^36^.

Here, we optimise these probes for expression in primary CTLs and demonstrate that they exhibit biased localisation during migration without affecting migration speed or CTL function. We perform traction force microscopy (TFM) on CTLs expressing utrn-CH-based actin conformation probes during both migration and synapse formation. Inhibition of non-muscle myosin IIa, formins, or Arp2/3 is shown to disrupt force generation and actin structures, but not biased probe localisation, indicating upstream regulation of actin conformation in cells. Dynamic changes to actin conformation at the cytotoxic immune synapse, both on TFM gels and in CTL-target conjugates, revealed a compression-associated conformation dominating the early cytotoxic immune synapse, and a tension-associated conformation ensuing later on. Notably, force production over time mimicked the pattern of compression-associated F-actin conformation at the synapse, but with a time lag of ~60 seconds. Thus, dynamical actin conformation is both regulated upstream of force production and temporally precedes force exertion in CTLs. Our work expands understanding of forces generated by T cells by focusing on primary CTLs (rather than cell lines), and presenting insight into actin conformation as it correlates with force production during both migration and killing.

## Results

### Actin conformation probes exhibit biased localisation in migrating primary CTLs and do not affect cell function

**Fig. 1:**
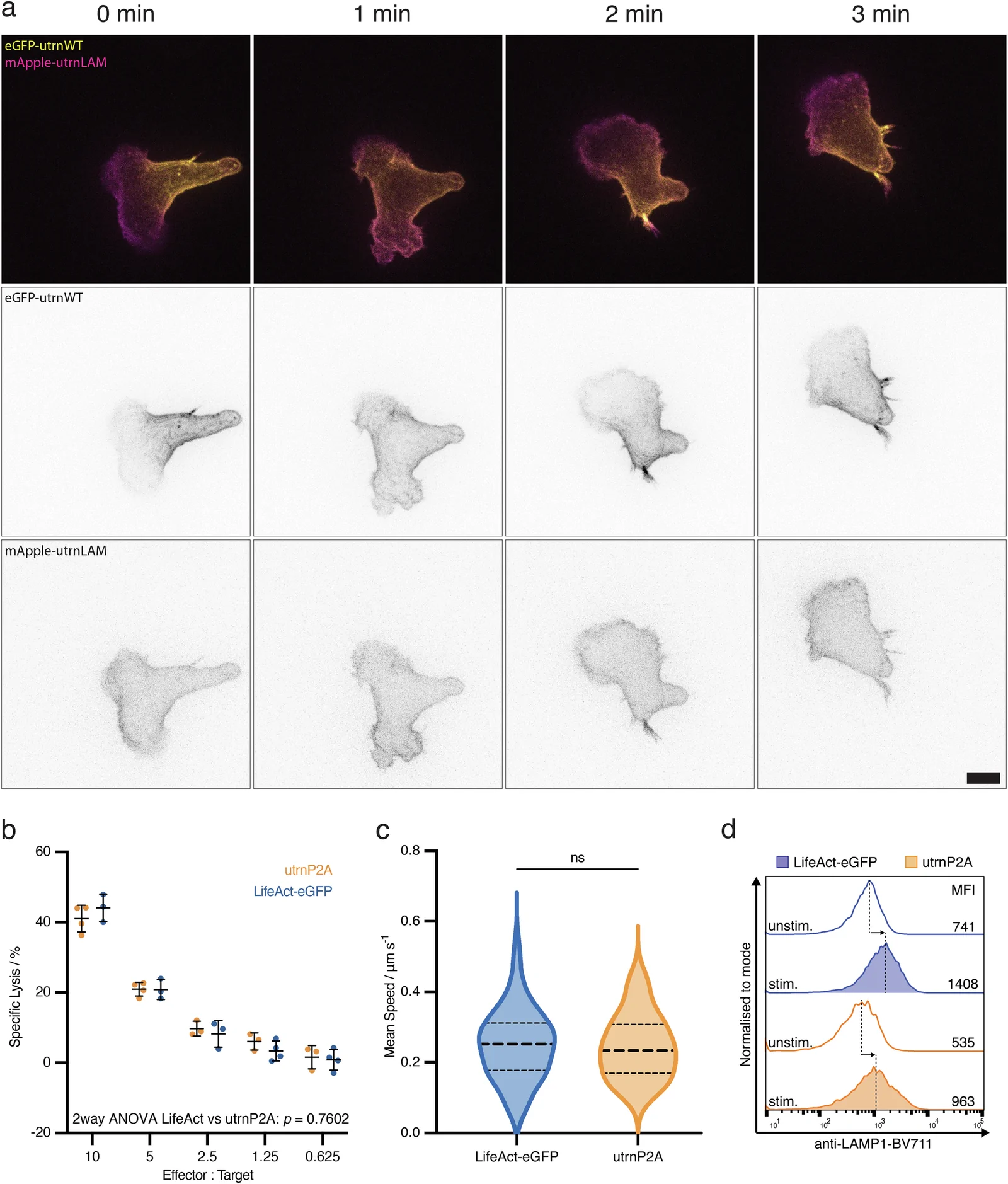
Actin conformation probes do not affect CTL function. **a** Time-lapse spinning disc confocal microscopy of an OTI CTL crawling on ICAM1-coated glass. The cell is expressing eGFP-utrnWT (yellow in composite) and mApple-utrnLAM (magenta in composite) from the utrnP2A construct. Images are maximum intensity projections. Separate channels are shown in inverted greyscale as indicated. Scale bar represents 5 μm. Representative of at least 30 cells from at least five independent experiments. **b** LDH killing assay comparing the ability of OTI CTLs expressing LifeAct-eGFP (blue) or utrnP2A (orange) to kill EL4 target cells at the indicated effector-to-target ratios. Representative of three independent experiments, bars represent the mean and standard deviation. **c** Migration assay showing mean migration speed of OTI CTLs expressing LifeAct-eGFP or utrnP2A crawling on ICAM1-coated glass. Horizontal dotted lines represent the median and interquartile range. Conditions were compared via an unpaired two-sided *t* test (LifeAct mean = 0.2502 μm/s; utrnP2A mean = 0.2453 μm/s; *p* = 0.7368). Representative of three independent experiments. **d** Degranulation assay based on cell surface exposure of secretory lysosome protein LAMP1 in response to TCR activation. Median fluorescence intensity for anti-LAMP1-BV711 in each case is given. The rightward shift upon stimulation is comparable between LifeAct-eGFP (blue) and utrnP2A (orange). Representative of three independent experiments.

To generate equal expression of utrnWT and utrnLAM probes in primary CTLs, we combined separate constructs into a single plasmid, using a P2A sequence^37^, and optimised fluorescent protein choice such that photobleaching would not significantly impact the fluorescence ratio between the two probes when imaging over time (Supplementary Fig. 1). Imaging CTLs transiently expressing these probes (“utrnP2A”) crawling on glass functionalized with the cell surface adhesion molecule ICAM-1 revealed a biased localisation, with a relative enrichment of utrnLAM versus utrnWT at lamellipodia and filopodial tips (Fig. 1a, Supplementary Movie 1). This indicated varied actin filament conformation across the cytoskeleton of migrating CTLs and was consistent with what has been shown in immortalised neutrophil-like cells^36^.

We next sought to validate that CTL function was unaffected by their expression. Using LifeAct-eGFP as an alternative commonly used F-actin probe for comparison^33^, we found no detrimental impact of utrnP2A expression on CTL-mediated killing, degranulation, or migration speed (Fig. 1b–d). Thus, utrnP2A could be reliably used to label areas of altered actin conformation in primary CTLs without affecting their function.

### Specific actin conformations are associated with but not sufficient for migratory force

To visualise force exertion by CTLs, we turned to TFM. Adapting a previously published protocol^38^, we synthesised polyacrylamide hydrogels by polymerising an acrylamide mixture directly onto pre-cleaned and treated commercially available live imaging dishes with microbead embedding via inverted polymerisation (Supplementary Fig. 2a, see Methods). Atomic force microscopy was used to measure gel stiffness and validate intra- and inter-batch consistency (Supplementary Fig. 2b, c). The resulting hydrogels exhibited an average Young’s modulus of 2.17 kPa (Supplementary Fig. 2b), which matches the stiffness of a typical cancerous APC^39^. Microbead distribution was even and appropriately spaced for high-resolution TFM of primary CTLs (Supplementary Fig. 2e).

**Fig. 2:**
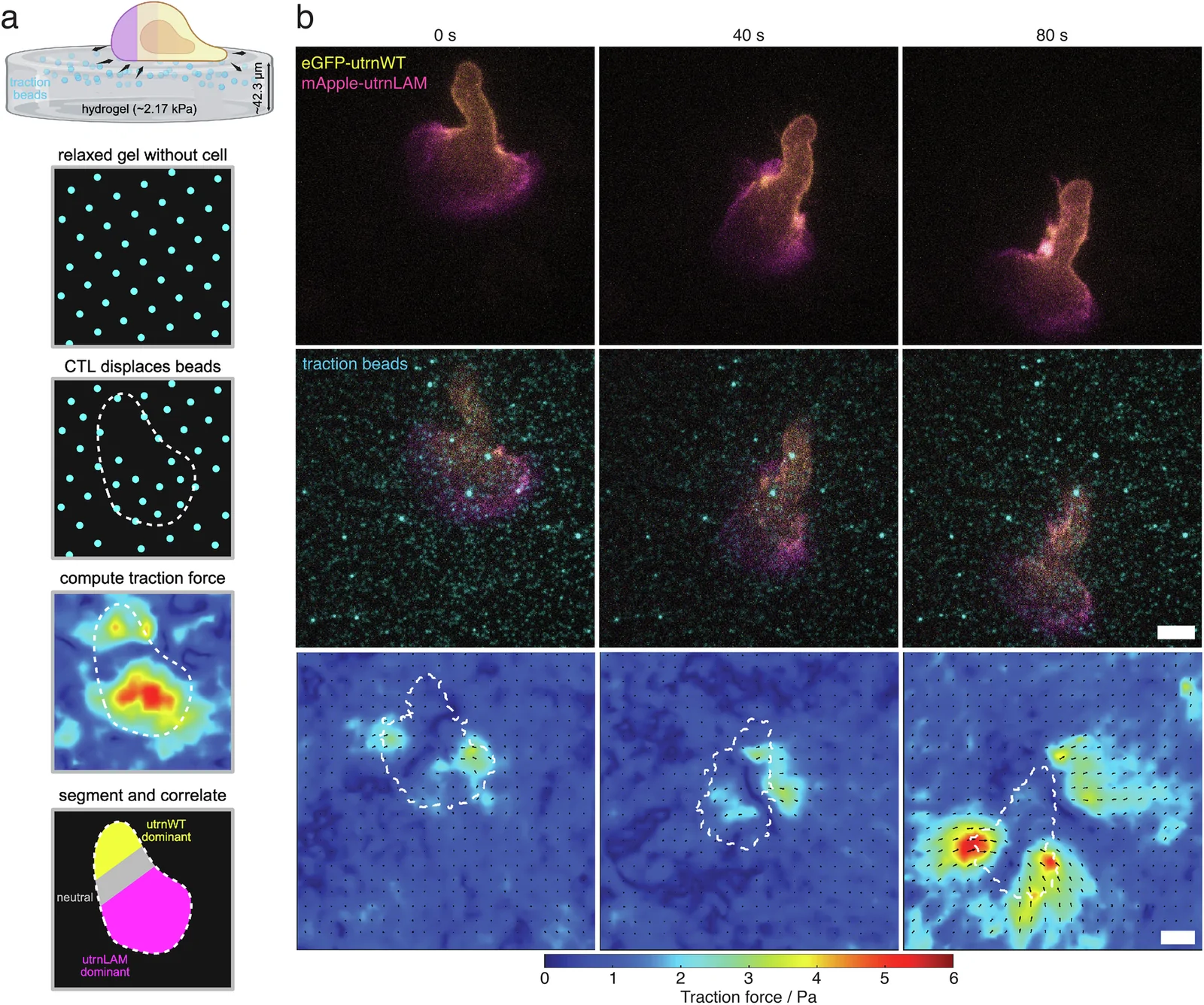
Traction force microscopy of migrating CTLs expressing actin conformation probes. **a** Schematic depicting traction force microscopy setup and analysis pipeline. Created in BioRender (https://BioRender.com/lq4jhqh).**b** Maximum intensity projection (top), single confocal slice in the plane of the TFM gel (middle), and traction force maps (bottom) of an OTI CTL expressing utrn probes (eGFP-utrnWT, yellow; mApple-utrnLAM, magenta) crawling on an ICAM-coated TFM gel (AF647 microspheres, cyan) with 2.17 kPa Young’s modulus. Scale bars represent 5 μm. Traction force is indicated by a colour scale between 0 (blue) and 6 Pa (red), with directional arrows shown. Representative of 40 cells across seven independent experiments.

Coating the TFM gels with ICAM-1, we seeded primary CTLs expressing utrnP2A and imaged their migration over time with a spinning disc confocal microscope (Fig. 2a). This revealed that biased localisation of actin conformation probes was maintained on soft hydrogels (Supplementary Movie 2, Fig. 2b). Using an established MATLAB script^40^, we analysed the bead displacement and calculated traction force exerted by CTLs on the TFM gel (Fig. 2a, b). We found strong (~6 Pa), forward-directed traction forces at the leading edge of the crawling CTLs, consistent with the notion of forceful branched F-actin polymerisation in lamellipodia during this type of migration^8^.

To understand whether force generation is dependent on actin conformation or vice versa, we perturbed the ability of CTLs to produce forces via three distinct pathways: (i) paranitroblebbistatin (pNB) was used to inhibit non-muscle myosin IIa, a motor protein known to play a role in maintaining polarity of migrating cells, maturation of adhesion foci, and enhancing leading edge protrusion^41–43^. (ii) CK666 was used to inhibit the Arp2/3 complex, which drives membrane protrusion during migration via actin branching, especially in lamellipodia. Arp2/3 is known to be mechanosensitive^44,45^. (iii) SMIFH2 was used to inhibit formins, which are a group of proteins sharing formin homology domains that are responsible for linear actin polymerisation^46^. Formins are a diverse group of actin polymerisation proteins that have been linked to both migration and immune synapse formation^27,47–49^.

**Fig. 3:**
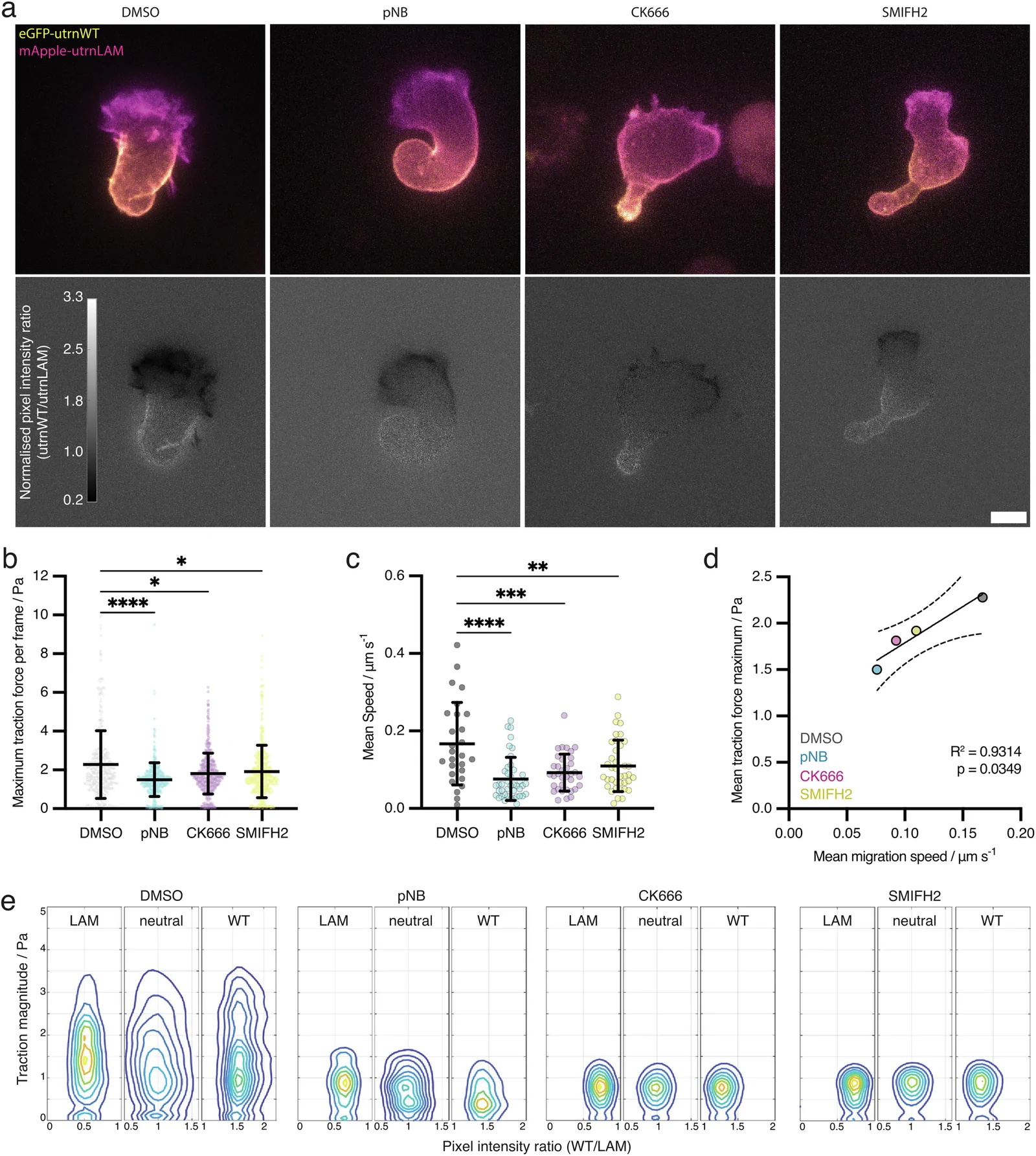
Perturbation of cytoskeletal machinery reduces migratory speed and force, but does not prevent biased probe localisation. **a** Maximum intensity projections of single time points from time-lapse spinning-disc confocal microscopy of CTLs crawling on ICAM1-coated TFM gels. CTLs are expressing utrnP2A (eGFP-utrnWT, yellow, and mApple-utrnLAM, magenta). Ratiometric images are shown in the bottom row, where a low value (dark) indicates utrnLAM dominance. Separate channels are shown in Supplementary Fig. 3. CTLs were treated with the indicated drugs. The scale bar represents 5 μm. **b** Comparison of maximum traction force per frame per cell between different drug treatments. Bars represent the mean and standard deviation. Conditions were compared via Kruskal–Wallis ANOVA with Dunn’s multiple comparisons test: DMSO (mean = 2.28 Pa, *n* = 369) vs pNB (mean = 1.50 Pa, *n* = 420) *p* < 0.0001; DMSO vs CK666 (mean = 1.81 Pa, *n* = 492) *p* = 0.0142; DMSO vs SMIFH2 (mean = 1.92 Pa, *n* = 465) *p* = 0.0128. **c** Comparison of migration speed on TFM gels. Mean cell speed was analysed using the TrackMate plugin in ImageJ and calculated in μm per second. Data points represent tracks from single cells. Mean and standard deviation are shown. Conditions were compared via one-way ANOVA with Dunnett’s multiple comparisons test: DMSO (mean = 0.1671 μm/s, *n* = 27) vs pNB (mean = 0.076 μm/s, *n* = 42) *p* < 0.0001; DMSO vs CK666 (mean = 0.0925 μm/s, *n* = 35) *p* = 0.0001; DMSO vs SMIFH2 (mean = 0.1099 μm/s, *n* = 38) *p* = 0.0036. Data are representative of three independent biological replicates. **d** Correlation between maximum traction force per frame and mean migration speed per track. Data points are the means across all data points and all experiments for each of the four drug treatments corresponding to the central horizontal lines in (**b**, **c**). DMSO = grey, pNB = cyan, CK666 = magenta, SMIFH2 = yellow. Simple linear regression was performed with a best-fit line shown (y=7.811x+1.008) in solid line and 95% confidence intervals in dotted curves. *R*^2^ and *p* values are shown. **e** Pixel distribution of traction magnitude segregated by probe ratio from all time points of representative cells in each drug treatment condition. “LAM” shows traction force at pixels with a utrnWT/utrnLAM ratio smaller than one standard deviation below 1. “neutral” shows the same for ratios between one standard deviation below and one standard deviation above 1. “WT” shows the same for ratios greater than one standard deviation above one. All data are representative of three independent experiments with 21 (DMSO), 14 (pNB), 17 (CK666), and 19 (SMIFH2) cells each across 12 frames of time-lapse imaging.

CTLs expressing utrnP2A were treated with each drug, seeded onto ICAM-1-coated TFM gels as before, and imaged over time. In DMSO-treated CTLs, lamellipodia and filopodia can be clearly seen, similarly to when migrating on glass (Fig. 3a, Supplementary Fig. 3a, Supplementary Movie 2). With pNB treatment, leading-edge lamellipodia are still present, but the rear of the cell is rounded and misshapen (Fig. 3a, Supplementary Fig. 3a, Supplementary Movie 3), as has been observed by others with blebbistatin-treated cells^50^. CK666-treated CTLs were unable to form lamellipodia, but instead formed filopodial protrusions at the leading edge as seen previously^51^, with enhanced relative utrnLAM binding (Fig. 3a, Supplementary Fig. 3a, Supplementary Movie 4). Finally, formin inhibition abrogated filopodia formation (Fig. 3a, Supplementary Fig. 3a, Supplementary Movie 5). In each instance, regions of F-actin with biased probe localisation were persistently observed, despite an inability to maintain cell shape, form lamellipodia, or form filopodia, respectively. Thus, biased localisation of F-actin conformation is regulated upstream or independently of actin-network structure (e.g., utrnLAM enrichment at the leading edge of migrating cells regardless of lamellipodial presence).

Analysing the force produced by migrating CTLs treated with our suite of inhibitors revealed a significant loss of migratory forces with all drug treatments, with the most significant from pNB treatment (Fig. 3b). Migration speed was similarly impacted by drug treatments, and force production and migration speed were found to be strongly positively correlated in migrating CTLs (Fig. 3c, d).

As with untreated cells, forward-directed traction forces from the utrnLAM-dominant lamellipodia were observed with DMSO-treated cells (Supplementary Fig. 3d). To determine the relative contributions of the different actin conformations to force production, we created a ratiometric channel (Fig. 3a) and segmented cells based on the ratio of probe expression in each pixel. Force production was found to predominantly occur in utrnLAM-dominant areas of the cytoskeleton of DMSO-treated cells, and, whilst regions of utrnLAM or utrnWT dominance persisted with drug treatments, this actin-conformation-correlated force production was abrogated with all drug treatments (Fig. 3e, Supplementary Fig. 3b, c).

Taken together, these data show that, on ICAM1-coated surfaces, migratory forces and speed depend on non-muscle myosin IIa, Arp2/3, and formins, with none being dispensable. Further, inhibition of these cytoskeletal elements and their characteristic actin reduces force production specifically in utrnLAM-dominant areas of F-actin, without impacting the biased localisation of actin conformation. This suggests that altered actin conformation is generated by the cell upstream of specific actin network structures and force production.

### Force-associated actin conformation precedes force exertion at the immune synapse

**Fig. 4:**
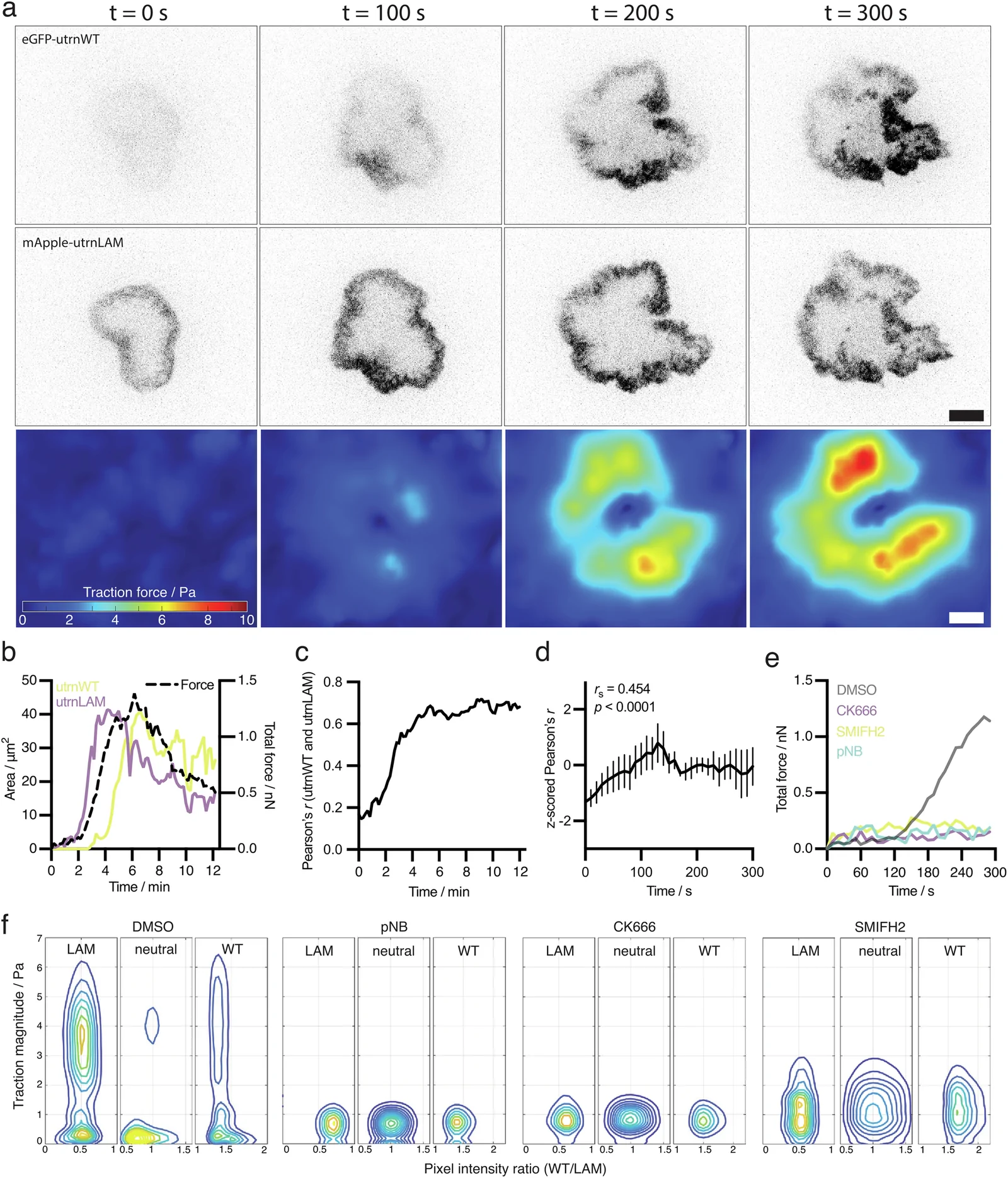
Dynamic changes to actin conformation at the cytotoxic immune synapse correlate with and precede force. **a** Single confocal slices of a representative primary CTL, expressing utrn probes (merged images shown in Supplementary Fig. 3) forming a synapse with an anti-CD3ε-coated TFM gel. The first time point shown is the point of initial contact between the CTL and the gel. Traction force maps for each of the four time points depicted are also shown (bottom row), with force in Pa. **b** Analysis of probe area and traction force, relating to (**a**). Left axis: area of eGFP-utrnWT (yellow) and mApple-utrnLAM (magenta) over time during artificial synapse formation. Areas were calculated by performing automatic Otsu thresholding on the time-stack per channel and applying it to each frame. Right axis: total force production over time in nN. **c** Co-localisation analysis through time between eGFP-utrnWT and mApple-utrnLAM in the representative traction force synapse shown in (**a**). **d** Co-localisation analysis for all control CTL replicates (*n* = 4). The zero time point was set to the point of initial contact of each cell with the TFM gel. Two-tailed Pearson’s correlation coefficients between the two probe channels were standardised for each replicate. Lines and errors depict the mean and SEM. A positive trend for co-localisation was tested via Spearman’s correlation. **e** Total force production, in nN, over time by representative CTLs, expressing utrnP2A, treated with cytoskeletal inhibitors, forming synapses with TFM gels. Scale bars represent 5 μm. **f** Pixel distribution of traction magnitude segregated by probe ratio for all time points from representative cells in each drug treatment condition (relating to **d**). All data are representative of 15 cells across three independent experiments.

Next, we turned to the immune synapse, where a high degree of force exertion has been observed^52,53^. We imaged primary CTLs expressing utrnP2A coming into contact and forming synapses with TFM gels functionalized with anti-CD3ε. A distinct spatiotemporal pattern of utrnWT and utrnLAM probe distribution was consistently observed. The initial contact included F-actin predominantly bound by utrnLAM that quickly depleted across the centre, forming an actin ring (*t* = 0 s, Fig. 4a, Supplementary Fig. 3e, Supplementary Movie 6). As the synapse progressed, utrnWT-bound F-actin was observed accumulating inside the outer ring of utrnLAM-dominant F-actin (*t* = 100 s, *t* = 200 s), and this proceeded to recover across the centre of the synapse as the synapse matured (*t* = 300 s, Fig. 4a, Supplementary Fig. 3f). Direct observation of bead displacement showed an inwardly directed force by the CTLs on the TFM gel (Supplementary Fig. 3e). This “pinching” by the actin-rich perimeter of the synapse is consistent with 2D TFM of Jurkat T cells^52^.

Primary CTLs exerted significant traction force at the synapse, peaking at ~10 Pa by 300 s (Fig. 4a, Supplementary Movie 7). By plotting total force production and probe area over time, we determined that force correlated with utrnLAM distribution with a ~ 50–100 s phase lag: The area of utrnLAM-bound F-actin rose steeply at 100 s, peaking near 40 μm^2^ at ~250 s, before falling gradually as utrnWT-bound F-actin increased in area. Concurrently, total force rose sharply beginning at ~150 s, peaking above 1 nN after 300 s, before falling gradually (Fig. 4b). This dynamic pattern of initial utrnLAM dominance at the synapse followed by utrnWT minutes later was detectable via co-localisation analysis between the two probes over time (Fig. 4c). In the cell shown, Pearson’s *r* between the two channels began under 0.2. Four minutes after synapse formation, this correlation had risen to above 0.6, where it remained for the duration of the experiment. This pattern was highly consistent, with the co-localisation between probes at the synapse showing a significantly positive trend over time across replicates (*r*_s_ = 0.454, *p* < 0.0001) (Fig. 4d). CTLs treated with cytoskeletal inhibitors, as before, were unable to produce force at the immune synapse (Fig. 4e), whilst regions of biased probe localisation persisted as in our migration experiments (Fig. 4f). The phase lag of force after utrnLAM-bound F-actin is further evidence that actin conformation is regulated upstream of force production, as shown by our TFM data of migrating CTLs treated with drugs (Fig. 3). These data, showing force production at the synapse is preceded by utrnLAM-associated F-actin conformation dominance, imply that dynamical actin filament conformation is necessary but not sufficient for force exertion at the synapse.

**Fig. 5:**
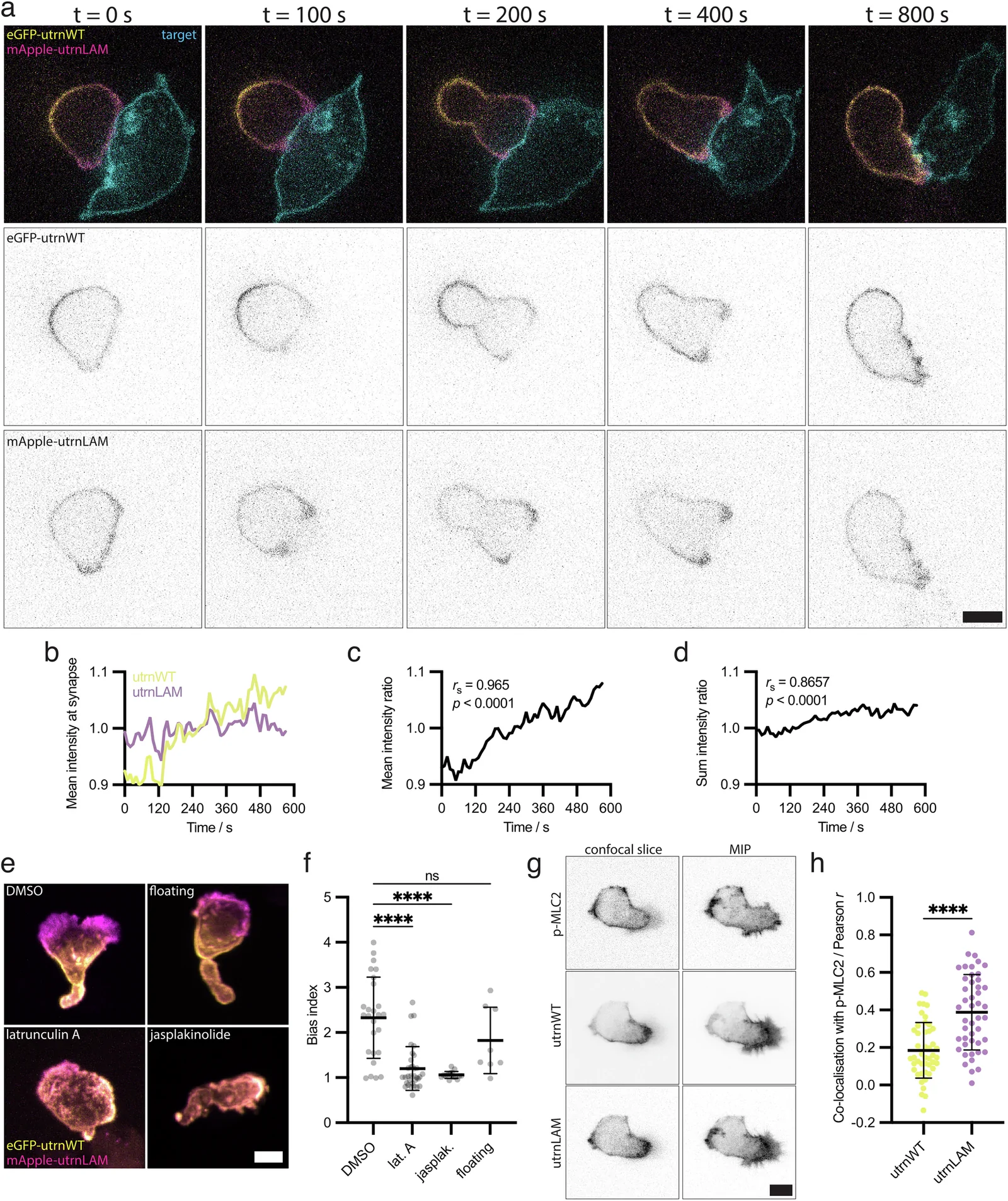
Polarised actin conformation is dynamic at the cell-cell immune synapse and is associated with phospho-myosin. **a** Time-lapse spinning-disc confocal microscopy of a representative primary OTI CTL, expressing utrnP2A (in merged images: eGFP-utrnWT in yellow, mApple-utrnLAM in magenta) forming an immune synapse with an EL4 target expressing membrane-targeted Electra2 (cyan) that had been pulsed with OVA_257–264_ peptide. The first time point shown is the point of initial contact between CTL and target. Single confocal slices are shown. Representative of 15 cells from across three independent experiments. **b**–**d** Dynamic probe intensities at the synapse over time. **b**, **c** Manual ROIs were drawn over the immune synapse of MIP images relating to (**a**). **d** A 3D volume of the synapse was segmented automatically in Imaris. Normalised probe mean intensities (**b**), the ratio of those mean intensities (**c**), and the ratio of summed 3D intensities (**d**) over time are shown. Trends over time were statistically tested with Spearman’s rank-order correlation. **e**, **f** Polarised F-actin conformation is present without tactile input but is abolished by latrunculin A and jasplakinolide. **e** Composite maximum intensity projections of representative cells expressing utrnP2A as above, either treated with DMSO (1:1000), latrunculin A (1 μM), or jasplakinolide (1 μM), or untreated and floating in solution. **f** Ratio of normalised probe intensities (LAM/WT) in the front/rear of the cell (“bias index”) across conditions in (**e**) Conditions were compared via ordinary one-way ANOVA with Dunnett’s multiple comparisons test: DMSO (mean = 2.326, *n* = 26) versus latrunculin A (mean = 1.202, *n *= 33) *p* < 0.0001; DMSO versus jasplakinolide (mean = 1.060, *n* = 16) *p* < 0.0001; DMSO versus floating (mean = 1.825, *n* = 8) *p* = 0.1420. **g**, **h** Phospho-myosin colocalises with utrnLAM more than utrnWT. **g** Single channel images of phospho-MLC2 (ser19) (AF647), eGFP-utrnWT, or mApple-utrnLAM. A single z-slice (in focus with the glass surface) and MIP are shown. **h** Colocalisation between p-MLC2 and utrnWT or utrnLAM, determined via the two-way Pearson correlation coefficient. Conditions were compared via a paired *t* test (*n* = 46): utrnWT (mean = 0.1848) versus utrnLAM (mean = 0.3878) *p* < 0.0001. Scale bar represents 5 μm. Data points represent individual cells pooled from three biologically independent replicates. Error bars represent means and standard deviations.

To test whether these striking dynamic changes to actin conformation at the cytotoxic immune synapse were also observed at cell-cell synapses potentiated by physiological TCR:pMHC interactions, we mixed target cells, presenting cognate antigen and expressing membrane-targeted Electra2, with primary CTLs expressing utrnP2A. Time-lapse 3D confocal microscopy showed a highly consistent pattern, with a utrnLAM-dominant lamellipodial leading edge making initial contact and forming an actin ring (Fig. 5a, Supplementary Movie 8 and 9). F-actin at the synapse was dominated by utrnLAM for the first ~200 s of the synapse, before actin conformation was altered, as seen by increased binding of utrnWT, culminating in complete recovery of cortical actin at the synapse by 800 s (Fig. 5a–d, Supplementary Movie 8, 9). The temporal dynamics of actin conformation between physiological CTL:target conjugates and artificial TFM synapses were directly comparable, validating the physiological relevance of our data.

### Actin conformation spatially correlates with phosphorylated myosin

Intrigued by the fact that CTLs exhibit biased utrn probe localisation before coming into contact with both traction force gels and cancerous targets, we sought additional ways to disrupt this bias. By imaging cells floating in solution above the glass surface, we confirmed that CTLs establish polarised actin conformations independently of tactile input (Fig. 5b, c). Next, we treated CTLs with the actin depolymerisation agent latrunculin A and the actin polymerising and stabilising drug jasplakinolide. In both instances, we found that the biased probe localisation along the front-rear axis of migrating cells was abolished (Fig. 5b, c). As latrunculin A treatment leads to actin depolymerisation, the loss of probe localisation is not surprising. Treatment with jasplakinolide, on the other hand, is known to stabilise actin states—trapping the actin conformation in a “young”, phosphate-bound state and possibly affecting interactions with actin-binding partners^54^. The loss of preferential binding shows that the utrophin probes are binding the actin conformation stabilised by jasplakinolide similarly, with jasplakinolide disrupting the actin conformation distribution found in untreated cells. These findings imply an active process, potentially driven by motor protein-derived tensions, by which the cells establish distinct actin conformations in different areas of the cytoskeleton.

To gain further insight into the molecular components involved, we fixed migrating CTLs expressing utrnP2A and stained them for myosin light chain 2 phosphorylated at serine-19 (p-MLC2). We found p-MLC2 present at the rear of the cells, where it is thought to drive contraction of the actin cytoskeleton that propels migrating T cells^55^, and where both utrn probes bind to F-actin similarly (Fig. 5d). However, we also found considerable p-MLC2 localisation at the leading edge of migrating CTLs, and thus it was colocalised significantly more with utrnLAM than utrnWT across the cells (Fig. 5d, e). These data present observational evidence that non-muscle myosin II may be involved in the establishment of distinct actin conformations across the cytoskeleton.

## Discussion

By combining TFM of CTL migration and immune synapse formation with probes sensitive to actin conformation, we could observe the spatiotemporal correlation of actin conformations with force outputs by the cells. Force was found to be produced predominantly from regions of the cytoskeleton dominated by utrnLAM, whilst the existence of these regions alone was not sufficient to produce force, as shown via treatment with cytoskeletal inhibitors. This force-associated F-actin (bound by utrnLAM) predominated the early immune synapse, being shortly followed by powerful force production. A shifting of the actin conformation to utrnWT-bound F-actin coincided with reduced force output and actin recovery across the centre of the immune synapse. Moreover, these spatiotemporal dynamics of F-actin conformation were consistent between TFM experiments and cell-cell cytotoxic immune synapses. Finally, we found that p-MLC2 was spatially coincident with this force-associated utrnLAM-bound F-actin—implying a role for this actin motor protein in establishing distinct actin conformations within the cell. Our work explores a parameter of the actin cytoskeleton that is important in both migration and killing by CTLs.

The binding of utrnLAM to leading-edge F-actin during migration correlated with a forward-directed traction force, indicating compression of the actin filaments where utrnLAM binds. The idea that utrnLAM-bound F-actin is compressed, whilst utrnWT-bound actin is under tension, is consistent with the actin conformation changes we observed at the immune synapse, where an inward-directed pushing force was exerted by a utrnLAM-dominant F-actin ring, which then favoured utrnWT binding as cortical actin recovered across the centre of the synapse and force waned. Nonetheless, future work focusing on in vitro reconstitution will be required to elucidate the precise link between probe binding bias, actin conformation, and cytoskeletal forces in greater molecular detail. In particular, the potential for a spatially coordinated role of actin conformation in the recruitment of non-muscle myosin II will be an exciting future direction, particularly with respect to actinomyosin arcs at the immune synapse which are known to be formin-dependent^27^.

Our use of cytoskeletal inhibitors reaffirmed prior work on the importance of non-muscle myosin IIa, Arp2/3, and formins in T-cell migration and immune synapse formation. Although off-target effects can occur with pharmacological interventions, perturbing the cytoskeleton genetically via electroporation-mediated siRNA or CRISPR/Cas9 was not compatible with electroporation-based plasmid expression. Future work might consider using viral transductions or primary T cells derived from patients harbouring mutations in genes implicated in actinopathies, such as *WAS*^56^, as a means to perturb the cytoskeleton genetically without the need for electroporation.

That said, CK666 and pNB in particular have been shown to be highly specific in vitro and in vivo^57,58^, and our experiments with them allowed us to decouple actin network architecture from force production. Our data here showed that differential probe localisation was maintained even while different actin-based structures (such as lamellipodia or filopodia) and force production were disrupted. This was surprising and implies that actin conformation is regulated upstream of force production, and is perhaps required for recruitment of actin-binding proteins to manipulate F-actin in varied ways and exert force. Notably, since TFM can only measure forces external to cells, it is equally possible that exclusively internal forces (that do not transmit to the cell periphery) drive different filament conformations, in turn affecting utrn probe distribution.

While temporal ordering of events and disruption of force production with inhibitors go some way to elucidating the mechanistic causality here—suggesting that actin conformation is upstream of, but not sufficient for, force production—we were unable to determine whether differential actin conformation is itself necessary for force production. Future studies will be required for further mechanistic insight here.

Since TFM is limited to ex vivo settings, fluorescence-based force probes that could be used in vivo are highly sought after to allow for biophysical studies to be performed in their proper physiological settings. Given the remarkable similarities between our 2D TFM and 3D cell-cell immune synapses, utrophin-based actin conformation probes have potential as a useful tool to study cellular biophysics in diverse in vivo systems, where TFM-based cellular biophysics is impossible. Migration of CD4^+^ T cells in a confined in vitro 3D environment has recently been shown to be surprisingly mesenchymal in nature, with focal adhesions being required to pull and push T cells forward^59^. It would be interesting to see how utrophin probe-based actin conformation is distributed in this confined migration model and, ultimately, in vivo.

While we showed that broad cellular function is unperturbed by expression of utrnP2A relative to LifeAct-eGFP, we cannot exclude the possibility that binding of utrnWT or utrnLAM does not itself impact actin filament conformation or dynamics, as is known for LifeAct^60^. Perturbation of the system to enable observation is regrettably unavoidable, and the expression of any probe binding to actin could have competition effects with other endogenous actin-binding proteins. Here, further structural work and in vitro assays would be required to assess the mechanistic impact of utrophin probes binding to actin.

## Methods

All antibodies, drugs and dyes are found in the reagents table (see Table 1).

**Table 1:** Reagents used

Reagents	Name	Target	Effect	Working concentration	Source	Catalogue number
**Drugs**	CK666	Arp2/3	Inhibition	100 μM	SLS	182515
	(S)-4’-nitro-blebbistatin	Non-muscle myosin II	Inhibition	20 μM	Cayman Chemical	CAY24171
	SMIFH2	Formin FH2 domain	Inhibition	20 μM	Merck	344092
	Latrunculin A	G-actin	Sequestering	1 μM	Cayman Chemical	10010630
	Jasplakinolide	F-actin	Stabilising	1 μM	Cayman Chemical	11705
**Dyes**	**Name**	**Target**	**Use**	**Working concentration**	**Source**	**Catalogue number**
	Zombie Violet™ Fixable Viability Kit	Dead cells	Flow	1:400 (flow)	BioLegend	423114
**Antibodies**	**Target**	**Host species**	**Clone**	**Working concentration**	**Source**	**Catalogue number**
	CD3ε (mouse), activating	Syrian hamster	eBio500A2	1 μg/mL	ThermoFisher	14-0033-82
	LAMP1 (mouse), BV711 conjugated	Rat	1D4B	1:200 (flow)	BioLegend	121631
	phospho-MLC2	Mouse	R.179.1	1:200	ThermoFisher	MA5-15163
	Mouse IgG	Goat	polyclonal	1:400	ThermoFisher	A32728TR

For each reagent type, column headers are shown in bold font.

### Mice

Tg(TcraTcrb)1100Mjb Rag1tm1Bal/tm1Bal (MGI:3054907 and MGI:2448994 alleles, referred to as OTI) mice were bred on a C57BL/6 background. Mice were housed in University of Cambridge establishments in individually ventilated cages at room temperature between 21–24 °C, humidity of 55% ± 10% and a 12/12 light/dark cycle. Mice were provided with ad libitum normal mouse diet and water, sizzle nest bedding, and enrichment. Breeding and maintenance of transgenic mice were carried out under UK Home Office project licence PP5905963. This research has been regulated under the Animals (Scientific Procedures) Act 1986 Amendment Regulations 2012 following ethical review by the University of Cambridge Animal Welfare and Ethical Review Body. ARRIVE reporting guidelines have been followed.

Spleens were obtained from male and female mice aged between 12 and 30 weeks. OTI mice were used because they offer an easy source of highly uniform CD8^+^ T cells with a known TCR.

### Plasmid design and cloning

For live fluorescence microscopy, the following plasmids were used:

**Table:** 

Construct	Labelled target	Source
LifeAct-eGFP	F-actin	Michael Davidson (Addgene #54610)^33^
utrnP2A	mApple-utrnLAM and eGFP-utrnWT	This study, adapted from ref. ^36^
mem-Electra2	Plasma membrane	This study

To design utrnP2A, the cDNA sequences of utrnLAM and utrnWT^36^, were N-terminally fused with mApple and eGFP respectively. A GSG linker followed by a P2A sequence^37^ was inserted 3’ of eGFP-utrnWT (with the stop codon removed), followed by mApple-utrnLAM. This construct was synthesised by Twist Biosciences (San Francisco, USA) with BamHI and NotI flanking sites, which were used to subclone into the pEGFP.N1 vector for transient expression in primary mouse T cells.

To design mem-Electra2, the first 20 amino acids of *Rattus rattus* neuromodulin^61^ were fused to the N-terminus of blue fluorescent protein Electra2 via a short linker^62^. The sequence was codon-optimised for *Mus musculus* and flanked with BamHI and NotI sites. The construct was synthesised by Twist Biosciences (San Francisco, USA) and subcloned into the pHR vector for lentiviral transduction.

### Cell culture

CTLs were from OTI mice, generated as previously described^63^. Briefly, OTI splenocytes were stimulated with 10 nM OVA_257-264_ peptide (SIINFEKL) (Cambridge Bioscience) in mouse T-cell medium: RPMI-1640 medium (SigmaAldrich, #1640) supplemented with 10% heat-inactivated FBS (LabTech, #FBS-SA), 50 mM β-mercaptoethanol (ThermoFisher, #31350010), 10 U/ml recombinant murine IL-2 (Peprotech, #212-12), 2 mM L-Glutamine (Sigma-Aldrich, #G7513), 1 mM sodium pyruvate (ThermoFisher Scientific, #11360070), and 50 U/ml penicillin and streptomycin (Sigma-Aldrich, #P0781). After three days, OVA_257-264_ was removed, and cells were thereafter maintained in mouse T-cell medium with daily replacement of medium via centrifugation and resuspension. Primary cells were incubated in a humidified atmosphere at 37 °C with 8% CO_2_.

Target cells for OTI CTL imaging experiments and killing assays were EL4 cells (ATCC: TIB-39; RRID: CVCL_0255) stably expressing mem-Electra2 (see below). Cell lines were maintained in DMEM (Sigma-Aldrich, #D5030) supplemented with 10% heat-inactivated FBS. Cell lines were incubated in a humidified atmosphere at 37 °C with 10% CO_2_.

### Lentiviral transduction

To generate EL4 target cells stably expressing mem-Electra2, Lenti-X 293 T cells (Takara, # 632180) were transfected with mem-Electra2 in the pHR backbone, pMD2.G (AddGene #12259), and pCMV ΔR8.2 (AddGene #12263) in a 5:1:4 ratio using TransIT reagent (Mirus Bio, #MIR 2704). Virus was harvested from the supernatant at 48- and 72 hours post-transfection, filtered, and concentrated using Lenti-X Concentrator (Takara, #631232). EL4 cells (ATCC: TIB-39; RRID: CVCL_0255) in logarithmic growth phase were resuspended in cell line medium at 4 × 10^6^/mL containing concentrated virus and polybrene at 1 μg/mL (Merck, #TR-1003-G). After 16 hours, the cells were diluted four-fold in cell line medium and cultured for three days before using FACS to isolate single-cell-derived clones based on Electra2 fluorescence.

### Nucleofection

OTI CTLs were nucleofected on days 4–6 post-stimulation from splenocytes. 5 × 10^6^ CTLs were washed in PBS via centrifugation (200 × *g*, 5 min) and resuspended, before final resuspension in nucleofection mix, comprised of 2.5 μg plasmid DNA and P3 Primary Cell Solution up to a total volume of 100 μL. Cells were electroporated in 100 µL Nucleocuvette™ vessels (Lonza, #V4XP-3024) with a 4D-Nucleofector® X unit (Lonza, #AAF-1003X) using pulse code DN-100. CTLs were immediately transferred, with micro-Pasteur pipette, to wells of a six-well plate containing 2 mL pre-warmed nucleofection recovery medium: calcium-free RPMI (US Biological, #R8999-02A), 5% heat-inactivated FBS, 2 mM L-glutamine, 32 μM 1-thioglycerol (Merck, #M6145), 1.7 mM sodium pyruvate, 20 μM bathocuproine disulfate (Merck, #B1125). CTLs were left to recover at 37 °C with 8% CO_2_ for 6 hours, before topping up with 6 mL of pre-warmed mouse T-cell medium.

### LDH killing assay

Killing of EL4 targets by OTI CTLs was determined by release of lactate dehydrogenase from lysed cells. Cells were seeded in round-bottomed 96-well plates at varied effector-to-target ratios, as indicated, in phenol red-free RPMI-1640 (ThermoFisher, #11835030) supplemented with 2% heat-inactivated FBS, and 50 U/mL penicillin and streptomycin. Plates were centrifuged for 5 minutes at 300 × *g* prior to incubation at 37 °C with 8% CO_2_ for three hours. Cell lysis was measured via CytoTox 96® Non-Radioactive Cytotoxicity Assay (Promega, #G1780). Briefly, after incubation, the plate was centrifuged once more and the supernatant transferred to a mirror-image flat-bottomed 96-well plate. Solubilised substrate mix (Promega, #G179A and #G180A) was added and incubated in the dark for 30 min, allowing for the enzyme-coupled reaction to proceed, resulting in conversion of tetrazolium salt into a red formazan product proportional to the number of lysed cells. After 30 min, absorbance at 490 nm was measured with a spectrophotometric plate reader. Specific target lysis was calculated by subtracting absorbance from medium-only control wells and comparing absorbance in wells containing effectors and targets with vs without OVA_257-264_ pulsing.

### Degranulation assay

CTLs were resuspended in fresh pre-warmed mouse T-cell medium at 1 × 10^6^ cells/mL. Fluorescently conjugated anti-mouse CD107a (LAMP1) (ThermoFisher, clone 1D4B, #12-1071) was added to the medium at 2 μg/mL, before seeding CTLs onto flat-bottomed plates that had been pre-coated with 1 μg/mL αCD3ε (‘stim.’) or PBS (‘unstim.’). Plates were incubated at 37 °C with 8% CO_2_ for the indicated time. Plates were then kept on ice and stained with a live-dead marker before analysing with an Attune NxT flow cytometer (ThermoFisher). The gating strategy is shown in Fig. S1c.

### Live imaging assays

CTLs were nucleofected with fluorescent protein constructs 24 hours prior to imaging. Prior to imaging, cells were resuspended at 1 × 10^6^ cells/mL in imaging medium: phenol red-free RPMI supplemented with 10% heat-inactivated FBS, 2 mM L-glutamine, 25 mM HEPES (ThermoFisher, #15630080), 50 U/mL penicillin-streptomycin.

For low-magnification migration speed assays: Imaging dishes (Mattek, #P35G-1.5-14-C) were pre-coated with 0.5 μg/mL ICAM-1 (R&D Systems, #796-IC) in PBS. 250 μL CTL suspension was added per dish and allowed 30 minutes at 37 °C with 8% CO_2_ for the cells to adhere and begin crawling. After this time, imaging medium was gently passed over the dish to remove non-adhered CTLs before imaging. Single confocal slices were captured with a ×20 objective lens (Leica HC PL APO ×20) in the mid-plane of the cells every 5 seconds for 5 minutes per field of view. Only the FITC channel was acquired (LifeAct-eGFP or eGFP-utrnWT). Three fields of view were taken per condition per experiment.

For high-magnification live imaging, CTLs were resuspended in imaging medium and dropped onto functionalized glass or TFM hydrogel. In the case of artificial synapses, cells were immediately imaged to capture them coming into contact with the activating surface. In the case of imaging migrating CTLs, cells were left to settle, adhere, and begin to crawl for 30 min prior to imaging through a ×100 objective lens (Leica HC PL APO ×100/1.40 OIL CS2). Confocal z-stacks with slices 0.8–1 μm apart were acquired over the whole volume of the conjugates every 10–30 seconds for up to 20 min.

For drug treatments, drugs were added to CTLs in imaging medium half an hour before imaging, and kept in throughout the assays. Working concentrations are given in the reagents table (Table 1). DMSO was used at a dilution of 1:1000.

In the case of imaging of conjugates, EL4 target cells expressing mem-Electra2 were pulsed with 1 μM OVA_257-264_ peptide for half an hour and then washed thrice by centrifugation and resuspension in cell line medium. Target cells were ultimately resuspended in RPMI (serum-free) at 1 × 10^6^ cells per mL, and 250 μL seeded onto imaging dishes that had been pre-coated with ICAM as before. After 10 minutes at 37 °C with 8% CO_2_, imaging medium was gently passed over the imaging dish to remove non-adhered target cells. Nucleofected CTLs were dropped onto target cells and allowed 10 minutes to settle before imaging.

All live microscopy used an Andor spinning-disk confocal system (Revolution; Andor) fitted with a CSU-X1 spinning-disk unit (Yokogawa) via a DMi8 microscope (Leica). Samples were excited with a combination of lasers at 405, 488, 561, and 637 nm wavelength. Images were captured using an iXon Ultra 888 camera and Fusion software (Andor). Imaging was performed with cells at 37 °C, 5% CO_2_ in an airflow sample chamber on the stage (OkoLab).

### Immunocytochemistry

To image p-MLC2, CTLs expressing utrnP2A were fixed by addition of paraformaldehyde to 4% and incubation at room temperature for 20 min. After washing with PBS, cells were permeabilised with 0.1% Triton X-100 for 10 min at room temperature. Cells were stained with an antibody against phospho-MLC2 (Ser19) (ThermoFisher, #MA5-15163) at a dilution of 1:200 in ICC buffer (PBS with 1% BSA) for one hour at room temperature. After washing, secondary staining was performed with an AF647-conjugated anti-mouse IgG antibody (ThermoFisher, #A32728TR) at a dilution of 1:400 in ICC buffer for 45 min at room temperature. Cells were washed and imaged in PBS without mounting.

### TFM gel preparation

Gel preparation was adapted from published work^38^ as follows:

For glass preparation: Imaging dishes (Mattek, #P35G-1.5-14-C) were cleaned via three 5 min incubations with 100% ethanol. After final aspiration, the glass was left to air-dry completely. The glass was then silanized via 30 min incubation at room temperature with 0.5% (3-aminopropyl)trimethoxysilane (APTMS) (Sigma-Aldrich, #281778) in ultrapure sterile water. APTMS solution was then aspirated, and the glass was rinsed with water thoroughly. The surface was prepared for gel adhesion by incubation for 30 min at room temperature with 0.25% glutaraldehyde (Sigma-Aldrich, #354400). The glass was rinsed thoroughly with water again and left to air-dry. Top coverglasses were prepared by cleaning 12 mm circular glass coverslips (VWR, #630-2200) via water bath sonication in 100% ethanol for 10 min, then leaving them to air-dry on a clean Kimwipe.

For gel preparation: Gel premix was created by adding 500 μL of 40% acrylamide solution (SLS, #A4058) and 65 μL of N-hydroxyethyl acrylamide (Sigma-Aldrich, #697931) to an Eppendorf tube and mixing. 65 μL was then removed (leaving 500 μL), and 250 μL of 2% N,N’-methylenebisacrylamide solution (Sigma-Aldrich, #M1533) was then added and mixed by vortexing. To create TFM gels with an average stiffness of 2.17 kPa, 65 μL of gel premix was added to 425 μL of DPBS (Gibco, #14190144) and mixed. To this, 10 μL of dark red 0.2 μm FluoSpheres™ Carboxylate-Modified Microspheres (ThermoFisher, #F8807) was added. This mixture was vortexed and then sonicated in a water bath sonicator for 10 min, and subsequently degassed via a vacuum desiccator for 4 min.

For TFM gel synthesis: To polymerise the gel, 1.5 μL of N,N,N’,N’-tetramethylethylenediamine (Sigma-Aldrich, #T9281) and 5 μL of 10% (w/v) ammonium persulfate solution (Sigma-Aldrich, #A3678) were added to 500 μL of TFM gel mix and mixed by gentle pipetting. As quickly as possible, 3 μL was added to the centre of the prepared glass imaging dish. A prepared top coverglass was then inverted on top, causing the gel to spread to the edges of the 12 mm glass coverslip. The entire imaging dish was then inverted and left for 15 min at room temperature for the gel to polymerise. Flipping the dish back again, DPBS was added to cover the glass and left for 30 min. Then the top cover glass was removed gently with tweezers. Gels were treated with poly-D-lysine (Gibco, #A3890401) overnight at 4 °C. The gel could then be functionalized with a biologically relevant molecule.

For migration on TFM gels, poly-D-lysine was rinsed three times with PBS, then replaced with 50 μg/mL ICAM-1(R&D Systems, #796-IC) in PBS and incubated at 37 °C for 1 hour, before three PBS washes and cell mounting. For artificial immune synapses on TFM gels, the same process was performed, but substituting ICAM-1 for anti-CD3ε (ThermoFisher, #14-0033-82) at the same concentration of 50 μg/mL. High concentrations were used to compensate for poor functional molecule adhesion compared to regular glass coverslips, ensuring saturation of the surface.

Gel thickness was measured on the microscope by focusing on the fluorescent beads on the top and bottom of the gel, subtracting the stage positions accordingly (Supplementary Fig. 2d).

### Image analysis

Migration assay data were analysed with ImageJ software using the TrackMate plugin. Cells were masked using a Laplacian of Gaussian detector, with an estimated diameter of 11 μm and threshold of 2.0. Migration was tracked between frames via a simple LAP tracker, with 5 μm maximum linking distance and zero tolerance for gaps. Tracks with fewer than 10 spots were excluded from analysis. Mean track speed was exported and plotted in Prism software (GraphPad).

TFM assays were analysed via MATLAB, customising a published script (GitHub: https://github.com/DanuserLab/u-inferforce^40^). For the TFM analysis of the bead movement, the slice of the confocal z-stack, where the surface of the gel was in focus, was selected. Frames were drift-corrected relative to each other by efficient subpixel registration, and bead displacement was calculated frame-to-frame. Using the empirically measured gel stiffness of 2.17 kPa, traction force was then calculated using Fourier transform traction cytometry, with the L-curve method employed for regularisation parameter selection, using L-optimal criteria. To calculate the amount of force in the cell area, a cell mask was created using the Otsu threshold method for both utrnWT and utrnLAM fluorescent channels. The utrophin masks were then summed up to generate a cell mask. The force in the cell area was calculated by overlaying the force field with the generated cell mask and summing the magnitudes of the force vectors inside the cell area.

To determine the probe ratio in the maximal force zone, a 30 px radius circle centred on the maximal force pixel was used to ascertain the average probe ratio in this area.

For TFM analysis based on utrn probe distribution, each frame was normalised to the maximum intensity pixel before histogram-matching in each probe channel. For ratiometric cell segmentation, the utrnWT/utrnLAM ratio was calculated and categorised as “utrnLAM dominant”, “utrnWT dominant”, or “neutral” based on a ratio value two standard deviations below 1, two standard deviations above 1, or between the two thresholds. For migrating cells, maximum intensity projections were used to create the ratiometric image. For synapses, only the bottom z-slice (in focus with the synapse) was analysed.

For analysis of utrn probe area over time at the synapse, we generated time-stacks of each probe in the plane of the gel and performed automatic Otsu thresholding (such that all timeframes were incorporated into the thresholding algorithm). Total area per probe for each frame was then calculated based on these thresholds.

For colocalization analysis between utrnWT and utrnLAM at the synapse, a time-dependent colocalization channel was generated in Imaris (version 11.0.1, Oxford Instruments) with dynamic automatic thresholding for each channel and time point. Pearson’s correlation coefficient for each time point was extracted. For analysis across replicates, these *r* values were z-scored for each movie. Spearman’s rank-order correlation was used to test for a trend in colocalization over time.

For analysis of probe bias in CTL-target conjugates, initially (Fig. 5b, c) manual ROIs were drawn over the synapse for each timepoint as a MIP and mean intensity for each probe was measured and then normalised across timepoints. Alternatively (Fig. 5d), a region of the CTL in close proximity to the target cell was generated in Imaris software by creating a generous segmentation surface based on target cell fluorescence and then segmenting CTL within that surface based on utrnLAM intensity. Sum intensities for each probe in 3D were then measured within that synapse-specific CTL volume (which moved across timeframes automatically). Spearman’s rank-order correlation was used to test for a trend in probe bias over time.

For analysis of probe bias in drug-treated and floating cells, boxes were manually drawn over the rear and the front half of migrating cells in ImageJ. The average intensities of each utrn probe were measured in each box and normalised to the whole-cell average per channel. The ratio of normalised probe intensities in the front and rear of the cells was then calculated and expressed as a “bias index” which represents utrnLAM enrichment at the front of the cell.

For p-MLC2 colocalization analysis, Pearson’s correlation coefficient was calculated in ImageJ between p-MLC2 and utrnWT or utrnLAM channels on a per-pixel basis.

Data from imaging of live conjugates were analysed and processed for export in Imaris software (BitPlane). Image channels were pseudo-coloured for optimal visual contrast.

### Atomic force microscopy

AFM measurements were performed on a JPK CellHesion200 (Bruker), with a tipless cantilever (ArrowTL, NanoWorld) to which a 37 μm diameter polystyrene bead (PS-R-37.0–microParticles GmbH) had been glued. The spring constant of the cantilever, determined by thermal tuning, was 0.092 N/m, the setpoint 10 nN and the speed of the Z scanner was 10 μm/s. Force-distance curves were analysed in the JPK Data Processing software (Bruker), where the Hertz-Sneddon Model was applied to calculate Young’s Modulus, assuming Poisson’s ratio to be 0.5.

### Data presentation

Data were processed, statistical tests applied, and data plotted in Excel (Microsoft) and/or Prism (GraphPad). Flow cytometry data were plotted in FlowJo (BD). Schematic diagrams were produced in BioRender. Figures were collated in Illustrator (Adobe). The manuscript was processed in Word (Microsoft).

### Statistics and reproducibility

Number of cells (*n*) and biologically independent replicates (*N*), specific statistical tests, and *p* values are given in figure captions. Statistical tests were performed in Prism (GraphPad). All tests were two-tailed.

### Reporting summary

Further information on research design is available in the Nature Portfolio Reporting Summary linked to this article.

## Supplementary information

Transparent Peer Review file

Supplementary Information

Description of Additional Supplementary Files

Supplementary Data

Supplementary Movie 1

Supplementary Movie 2

Supplementary Movie 3

Supplementary Movie 4

Supplementary Movie 5

Supplementary Movie 6

Supplementary Movie 7

Supplementary Movie 8

Supplementary Movie 9

Reporting Summary

## Data Availability

This work produced no large datasets. Tabular data for all graphs is available as Supplementary Data. Raw data will be shared upon request.
