# Supplementary material for: Actin conformation dynamics precede force generation in cytotoxic T lymphocytes
Source: Commun Biol. 2026 Sep 25;9:1256. doi: 10.1038/s42003-026-11011-3 (PMC13614940; doi:10.1038/s42003-026-11011-3)
Supplement: Supplementary file 1 [file 42003_2026_11011_MOESM14_ESM.pdf]

nature portfolio

Corresponding author(s): Prof Anna H Lippert, PhD

Last updated by author(s): Apr 8, 2026

Reporting Summary

Nature Portfolio wishes to improve the reproducibility of the work that we publish. This form provides structure for consistency and transparency in reporting. For further information on Nature Portfolio policies, see our Editorial Policies and the Editorial Policy Checklist.

Statistics

For all statistical analyses, confirm that the following items are present in the figure legend, table legend, main text, or Methods section.

n/a	Confirmed	
☐	☒	The exact sample size (*n*) for each experimental group/condition, given as a discrete number and unit of measurement
☐	☒	A statement on whether measurements were taken from distinct samples or whether the same sample was measured repeatedly
☐	☒	The statistical test(s) used AND whether they are one- or two-sided<br>*Only common tests should be described solely by name; describe more complex techniques in the Methods section.*
☒	☐	A description of all covariates tested
☐	☒	A description of any assumptions or corrections, such as tests of normality and adjustment for multiple comparisons
☐	☒	A full description of the statistical parameters including central tendency (e.g. means) or other basic estimates (e.g. regression coefficient) AND variation (e.g. standard deviation) or associated estimates of uncertainty (e.g. confidence intervals)
☐	☒	For null hypothesis testing, the test statistic (e.g. F, t, r) with confidence intervals, effect sizes, degrees of freedom and P value noted<br>*Give P values as exact values whenever suitable.*
☒	☐	For Bayesian analysis, information on the choice of priors and Markov chain Monte Carlo settings
☒	☐	For hierarchical and complex designs, identification of the appropriate level for tests and full reporting of outcomes
☐	☒	Estimates of effect sizes (e.g. Cohen's *d*, Pearson's *r*), indicating how they were calculated

Our web collection on statistics for biologists contains articles on many of the points above.

Software and code

Policy information about availability of computer code

Data collection	No software was used to collect data in this study.
Data analysis	Traction force microscopy analysis was performed using publicly available code published by others. GitHub: https://github.com/DanuserLab/u-inferforce.

For manuscripts utilizing custom algorithms or software that are central to the research but not yet described in published literature, software must be made available to editors and reviewers. We strongly encourage code deposition in a community repository (e.g. GitHub). See the Nature Portfolio guidelines for submitting code & software for further information.

Data

Policy information about availability of data

All manuscripts must include a data availability statement. This statement should provide the following information, where applicable:

- Accession codes, unique identifiers, or web links for publicly available datasets
- A description of any restrictions on data availability
- For clinical datasets or third party data, please ensure that the statement adheres to our policy

This work produced no large datasets. Tabular data for all graphs is available as Supplementary Data.

Research involving human participants, their data, or biological material

Policy information about studies with human participants or human data. See also policy information about sex, gender (identity/presentation), and sexual orientation and race, ethnicity and racism.

Reporting on sex and gender	n/a
Reporting on race, ethnicity, or other socially relevant groupings	n/a
Population characteristics	n/a
Recruitment	n/a
Ethics oversight	n/a

Note that full information on the approval of the study protocol must also be provided in the manuscript.

Field-specific reporting

Please select the one below that is the best fit for your research. If you are not sure, read the appropriate sections before making your selection.

☒ Life sciences ☐ Behavioural & social sciences ☐ Ecological, evolutionary & environmental sciences

For a reference copy of the document with all sections, see nature.com/documents/nr-reporting-summary-flat.pdf

Life sciences study design

All studies must disclose on these points even when the disclosure is negative.

Sample size	Sample size varied by assay throughput. Where possible, >30 cells were used. For low throughput microscopy assays, at least five and typically more than ten cells per conditions were imaged.
Data exclusions	Data were not excluded.
Replication	All experiments were replicated at least three times with biologically independent samples.
Randomization	This is not relevant for this study. Splitting cell populations into different treatment groups is inherently random.
Blinding	Blinding was not possible or relevant for this study.

Reporting for specific materials, systems and methods

We require information from authors about some types of materials, experimental systems and methods used in many studies. Here, indicate whether each material, system or method listed is relevant to your study. If you are not sure if a list item applies to your research, read the appropriate section before selecting a response.

Materials & experimental systems

n/a	Involved in the study	
☐	☒	Antibodies
☐	☒	Eukaryotic cell lines
☒	☐	Palaeontology and archaeology
☐	☒	Animals and other organisms
☒	☐	Clinical data
☒	☐	Dual use research of concern
☒	☐	Plants

Methods

n/a	Involved in the study	
☒	☐	ChIP-seq
☐	☒	Flow cytometry
☒	☐	MRI-based neuroimaging

Antibodies

Antibodies used	anti- mouse CD3ε; species: Syrian hamster; clone: eBio500A2; source: ThermoFisher; catalogue number: 14-0033-82<br>anti-mouse LAMP1 (BV711 conjugated); species: Rat; clone: 1D4B; source: BioLegend; catalogue number: 121631<br>anti-phospho-MLC2; species: Mouse; clone: R.179.1; source: ThermoFisher; catalogue number: MA5-15163

	Goat anti-Mouse IgG (H+L) Highly Cross-Adsorbed Secondary Antibody, Alexa Fluor™ Plus 647; clone: polyclonal; source: ThermoFisher; catalogue number: A32728TR
Validation	Stimulating anti-mouse CD3ε (RRID: AB_837128) has been validated for this application by the manufacturer and in independent publications (e.g.: https://doi.org/10.1126/science.abp8933).<br>Anti-mouse LAMP1 (RRID: AB_2783065) has been validated by the manufacturer for flow cytometry and in independent publications (e.g., https://doi.org/10.1016/j.celrep.2021.110021)<br>Anti-phospho-MLC2 (RRID: AB_10979087) has been validated by the manufacturer for ICC/IF and in independent publications (e.g., https://doi.org/10.1242/jcs.248732)

Eukaryotic cell lines

Policy information about cell lines and Sex and Gender in Research

Cell line source(s)	EL4 target cells were obtained from ATCC (catalogue no.: TIB-39; RRID: CVCL_0255)
Authentication	These cells were purchased directly from ATCC, who authenticated the cell line.
Mycoplasma contamination	We used cells for experiments within 20 passages. Mycoplasma testing was performed monthly if applicable. No contaminations were detected.
Commonly misidentified lines (See ICLAC register)	n/a

Animals and other research organisms

Policy information about studies involving animals; ARRIVE guidelines recommended for reporting animal research, and Sex and Gender in Research

Laboratory animals	Mice used were C57BL/6 (B6)-OTI Rag2−/− (genotype: B6.129S6-Rag2tm1Fwa Tg[TcraTcrb]1100Mjb). Animals used were male and female aged 12-30 weeks.
Wild animals	n/a
Reporting on sex	Males and females were used. No sex differences were detected in any of our data.
Field-collected samples	n/a
Ethics oversight	This research was regulated under the Animals (Scientific Procedures) Act 1986 Amendment Regulations 2012 following ethical review by the University of Cambridge Animal Welfare and Ethical Review Body (AWERB).

Note that full information on the approval of the study protocol must also be provided in the manuscript.

Plants

Seed stocks	n/a
Novel plant genotypes	n/a
Authentication	n/a

Flow Cytometry

Plots

Confirm that:

☒ The axis labels state the marker and fluorochrome used (e.g. CD4-FITC).

☒ The axis scales are clearly visible. Include numbers along axes only for bottom left plot of group (a 'group' is an analysis of identical markers).

☒ All plots are contour plots with outliers or pseudocolor plots.

☒ A numerical value for number of cells or percentage (with statistics) is provided.

Methodology

Sample preparation	Cells were primary mouse T cells that had been grown in cell culture for a period of days. For flow cytometry, live cells were stained on ice and kept at 4 degrees C for all steps and acquisition. These are uniform suspension cells, so no further processing is required beyond resuspension.
Instrument	ThermoFisher Attune NxT
Software	Acquisition: Attune NxT Software<br>Analysis: FlowJo version 10.
Cell population abundance	No sorting was performed. OTI T cell suspensions are a ~pure population o CD8+ T cells.
Gating strategy	Events were gated on FSC-A x SSC-A for lymphocytes, FSC-A x FSC-H for singlets, and FSC-A x VL1-A for live cells (using Zombie Violet live/dead stain). LAMP1 (BV711) fluorescence was assessed for cells in the Q1 gate of the BL1-A x YL1-A channel for LifeAct-eGFP-expressing cells and Q2 for utrnP2A-expressing cells. Compensation was not necessary. Further details in Figure S1.

☒ Tick this box to confirm that a figure exemplifying the gating strategy is provided in the Supplementary Information.
