# Supplementary material for: Actin conformation dynamics precede force generation in cytotoxic T lymphocytes
Source: Commun Biol. 2026 Sep 25;9:1256. doi: 10.1038/s42003-026-11011-3 (PMC13614940; doi:10.1038/s42003-026-11011-3)
Supplement: Supplementary file 2 [file 42003_2026_11011_MOESM1_ESM.pdf]

nature portfolio

Peer Review File

Actin conformation dynamics precede force generation in cytotoxic T lymphocytes

Corresponding Author: Professor Anna Lippert

This file contains all reviewer reports in order by version, followed by all author rebuttals in order by version.

Version 0:

Reviewer comments:

Reviewer #1

(Remarks to the Author)
The manuscript titled "Actin conformation dynamics precede force generation in cytotoxic T lymphocytes" by Rochussen AM et al., provides a valuable insight into the principles of force regulation within the CTL's cortex and the CTL-target interface, i.e., immune synapse. The authors performed a series of careful spatio-temporal analyses of utrophin-CH domain-based F-actin conformation probes to decipher the distribution of the compressed (utrnLAM binding) and tensed (utrnWT binding) filamentous actin.

The key findings are as follows:
1. F-actin conformational changes occur prior to the force generation. Thus, within the causality hierarchy, the actin conformation is upstream of the mechanisms that generate force. I think this is the most important and central finding of the presented work. This raises the question of whether there is a potential feedback regulatory mechanism that links actin conformation to the amount of force within the cytoskeleton.
2. Time coordination of the forces within the immune synapse. Specifically, the transition from compressed F-actin towards tensed F-actin is first observed in the form of the transition from utrnLAM dominance to utrnWT binding.
3. The similarities between artificial IS and the interactions between CTL and live cell targets demonstrate the physiological relevance of these systems.

The work demonstrates substantial experimental and analytical soundness, technical advancement in the dual-expression of F-actin conformation probes in primary TCLs, and a tight and comprehensive integration of the TFM, inhibitor treatments, and live imaging.

The minor criticism that authors may choose to address in the form of discussion points is as follows:

1. Lack of a fully established mechanistic causality: While actin conformation changes precede force generation, it remains unclear whether these changes are necessary and sufficient to drive force production, or merely correlate with upstream signaling events.
2. Although Utrn probes are seemingly optimized and shown not to impair CTL function, their competitive binding with endogenous actin-binding proteins may still subtly change cytoskeletal dynamics. A discussion of possible artifacts or validation with orthogonal probes (e.g., LifeAct mutants or new tension sensors) would strengthen confidence in the results.
3. Over-reliance on pharmacological treatments. Although blebbistatin (and its derivatives), as well as CK666, are considered relatively specific inhibitors, SmifH2 has numerous off-targets and side effects that cannot always be accounted for in the experiment and the resulting data. What suggestions can authors make in their discussion to address these concerns?
4. Can authors also directly explore the contractility state of the F-actin via immunofluorescent staining of the phospho-myosin (Ser19) and confirm that its spatial distribution matches the tensed F-actin? Even if the two pictures do not match, this would be a valuable exploration of the state of the active (under myosin-driven tension) and passive (hydrostatically induced) tension of the F-actin in the CTL's cortex. Note that this experiment is not mandatory, but it would be desirable for many readers and experts in mechanobiology to see.

Overall, I find this manuscript to be a timely and essential advancement of the immune cell mechanobiology, and I strongly recommend its acceptance and publication.

I find the statistical analysis to be substantial, valid, and adequate for the experimental data.
I also find the experimental data and the description of the experimental techniques to be detailed enough for reproducibility tests and challenging the data by other research groups.

Thank you.

Prof. Tabdanov

Reviewer #2

(Remarks to the Author)
The manuscript “Actin conformation dynamics precede force generation in cytotoxic T lymphocytes” examines the relationship between actin filament conformation and traction force generation in cytotoxic T lymphocytes. The authors use newly developed variants of fluorescent tags that bind to actin depending on the filament conformation and TFM to relate the development of tractions to filament conformations. The authors find that actin filaments with distinct conformations assemble differentially along the cell-surface contact zone and suggest that conformation changes are independent of and precede force generation. While the results are interesting, the traction force measurements, which form the bulk of the paper, are likely in error. The authors comment on the discrepancies between their observations and findings in the literature, but it is hard to explain a 3 order of magnitude difference and their reported values are inconsistent with traction forces measured on the same type of T cells in the literature. Additionally, the claim that drug inhibition leaves the differences in conformation intact but abolishes forces is not supported by any analysis (other than the single representative example in Supplementary Figure 3a). At a bare minimum, correlation analysis of utmWT and utmLAM for the different conditions would be necessary to make such a strong conclusion. The manuscript would require significant revisions to address these issues and some specific comments as listed below.

Some specific comments:

• The authors report forces that are orders of magnitude higher than reported in the literature. Primary CTLs typically exert forces in the range of 2-20 nN on substrates of stiffness 1.2-6 kPa (Pathni et al., 2022, PMID: 35371019) or micropillar arrays (Basu et al., 2016, PMID: 26924577; other papers from Huse lab). The authors report forces of >10 uN at the cytotoxic immune synapse, which is extremely high! The raw traction values (<10 Pa), though low, seem reasonable given the lack of ICAM-1 in this experimental setup. However, the total forces seem to be erroneously high. A back of the envelope calculation, assuming that the peak tractions are ~6-10 Pa and the cell area is ~100 um2, yields a total force of ~1-2 nN, which is consistent with findings in the literature. It would be informative to see the displacement maps as well. Even adherent cells which exert peak tractions that are 10x that of CTLs have total forces in the 10-100 nN range! The authors should revisit their analysis and revise their conclusions and discussion accordingly as such large discrepancies do not inspire confidence in their main results.

• What was the thickness of the hydrogels used? A simple back-of-the-envelope calculation results in a gel thickness of ~12 um when using 3 uL of the gel polymerization of the mix with an 18 mm coverslip. As per previous literature, this may result in a gel that is too thin and may allow the cells to feel the stiffness of the underlying glass substrate. The authors should comment on the reason this gel thickness was selected, and how this might influence (i) cell behavior, (ii) AFM measurements of gel stiffness, and (iii) any intensity-based ratiometric calculations made through the hydrogel.

• An important paper to cite is Caillier et al., 2024 (PMID: 38889096), where TFM is used to measure forces for T helper cells migrating on PA gels, and these forces are correlated with the localization of focal adhesion proteins such as talin, vinculin and paxillin.

• Can the authors comment on how the respective localization of utrnWT- and utrnLAM-bound F-actin structures would compare to the conventional “bullseye” understanding of the distribution of branched and bundled actin structures at the T cell immune synapse and during T cell migration? utrnLAM-bound F-actin localizes to the branched actin-rich lamellipodia. utrnWT-bound F-actin is present in an annular region of the IS typically associated with actomyosin arcs, but also to actin foci towards the center of the IS which are generated by Arp2/3. Given that localization of these F-actin conformation-specific constructs is upstream of Arp2/3 or formin activity, how does actin conformation affect the recruitment of these actin-binding proteins? Can the authors comment on these points in the discussion?

Comments on figures: main

Figure 1
• Why was LifeAct used for comparisons for CTL function? When assessing whether expression of an actin probe impacts CTL function, a more appropriate control would be to use cells transfected with a construct that expresses just one or both fluorophores of interest, and additionally, untransfected cells.

Figure 2
• As presented, the figure just shows an anecdotal example and therefore does not merit a figure by itself. The authors should either present some quantification (potentially of differences in tractions between front and rear of the cell or merge

the figure with Figure 3).

Figure 3
• What was the DMSO concentration used for these experiments?
• How is the quantity (maximum force in the frame) calculated in Fig. 3b (and 3d)? Traction stresses have units of Pa (or kPa) while force has units of Newtons. Is the quantity presented the peak force (which is traction stress x cell area?).
• Also, the peak values of tractions are in the range of <10 Pa. So how is the value in kPa obtained?
• The quantity "Mean traction force maximum" in 3d is not defined.
• Figure 3d should include a legend indicating the conditions corresponding to the different colored symbols for readability.
• Figure 3e & 4e: The 2D density plots are quite tricky to read and not straightforward to interpret. The authors should explore alternative visualizations that may be easier to interpret. Perhaps calculating a single value of median traction per region (utrnLAM/utrnWT/neutral) per cell, and then comparing these in a violin/swarm chart or paired dot plot?

Figure 4
• What is the explanation for the utm-WT localization being asymmetric, while the synapse is in general symmetric?
• What is the explanation for force being correlated with utrn-LAM localization instead of with utrn-WT? Is this only true for the cell shown or representative of the population? The example in Figure 4a shows that WT is highly enriched in an area of high traction, but this is not apparent in the contour plot. The authors should carry out a correlation or cross-correlation analysis to support this point.
• It is remarkable that the pixel-wise traction magnitudes of cells with inhibitors is ~1 Pa, which is essentially background levels (including in their own figure). Multiple studies have shown that blebbistatin does not abolish traction forces (Hui et al. MBoC 2014, Basu et al. Cell 2016) – so the finding that blebbistatin entirely abolishes traction forces is puzzling. The authors should comment on this. The authors should include a supplementary figure showing traction forces and utm-WT and utm-LAM accumulation (similar to Fig 4a) for the inhibitors.
• While Figure 4c clearly demonstrates the temporal offset between LAM accumulation and subsequent WT accumulation, it is not clear whether this is a general effect across the population. A temporal cross-correlation analysis for such intensity/traction curves would be necessary to make general conclusions (e.g. see de Jesus et al., Fig. 1-2 for a similar point).

Figure 5
• The authors should present summary statistics to demonstrate that conjugates also show similar conformation dynamics. It is unclear what point the authors are trying to make and whether any effect is statistically robust.

Comments on text:
Line 130: Typo in text, should be "at lamellipodia and filopodial tips". Currently listed the other way round.

Reviewer #3

(Remarks to the Author)
In light of the growing recognition of the importance of physical forces in T cell function, the authors report that cytotoxic T lymphocytes (CTLs) employ utrophin-based actin conformation probes to visualize F-actin structures and spatial distribution, thereby uncovering a correlation between actin conformation and force generation.
The confocal and TFM imaging data, including both still images and time-lapse videos, are of high quality and effectively support the authors' claims. The use of a P2A sequence to ensure equimolar expression of the two probe variants is technically sound, and the TFM gel system is well-designed, with consistent stiffness closely matching that of cancerous antigen-presenting cells. These methodological strengths add credibility to the conclusions drawn from the experimental results. However, further validation would be necessary before this system can serve as a robust ratiometric reporter of actin filament conformation in live cells, particularly as a potential alternative to widely used probes such as LifeAct.

Major concerns
1.The authors state that utrnWT preferentially binds to structures such as filopodia, while utrnLAM is enriched in lamellipodia. However, direct evidence demonstrating the selective binding of utrnWT and utrnLAM to filopodia and lamellipodia, respectively, is lacking. If feasible, in vitro reconstitution of branched versus linear F-actin could be performed to validate whether utrnWT and utrnLAM indeed preferentially associate with these distinct actin architectures.
2.Additionally, while the authors show that regions of dominant utrnWT or utrnLAM localization persist upon treatment with Arp2/3 and formin inhibitors, further clarity could be gained by extending the pharmacological analysis to a broader range of actin-disrupting agents. For instance, treatment with Latrunculin A, which depolymerizes F-actin, or jasplakinolide, which promotes actin polymerization and stabilization, could reveal whether differential probe localization is maintained under a broader range of cytoskeletal perturbations..
3.It would also be informative to compare probe localization patterns in drug-treated cells with those in the basal state (untreated and non-migratory conditions), to determine whether the observed differential localization is a specific response to cytoskeletal perturbation rather than a general feature of non-motile cells.

Version 1:

Reviewer comments:

Reviewer #1

(Remarks to the Author)
The revised manuscript, titled "Actin conformation dynamics precede force generation in cytotoxic T lymphocytes" addresses all raised concerns adequately, reaching the standards of publication. The presented experimental data are now well shaped, the statistical representation and evaluation adequately reflect the strength and rigor of the experimental results, and also support the hypothesis suggested by the authors.

1. The work has been strong and impactful to begin with, but my concerns were mostly about the statistical strength. I now find these concerns properly addressed.

2. The methodology is now better described, and the materials used are sufficiently well identified for reproducibility purposes. The added supplementary figure with the detailed visual and verbal description of the experimental procedure is raising the manuscript to the next level of transparency and experimental reproducibility.

3. I find the presented work important to the field, broadening and deepening our understanding of the T cell cortex mechanobiology, and I believe this publication will be a key to both basic science researchers of the T cell motility/mechanobiology and to the clinical researchers who seek to improve the T cell migrational/invasive properties.

4. The claims that the actin conformational changes are the causal factors for T cell cortex contractility states are novel, important, well presented, and timely for the field.

I generally enjoyed the improved manuscript detail and data presentation.

Reviewer #2

(Remarks to the Author)
I have carefully reviewed the revised manuscript and the authors' point-by-point response. I am satisfied with the revisions made, as the authors have addressed all of my previous concerns and queries comprehensively. The manuscript has been significantly strengthened, and I have no further comments or suggestions for improvement at this stage.

Open Access This Peer Review File is licensed under a Creative Commons Attribution 4.0 International License, which permits use, sharing, adaptation, distribution and reproduction in any medium or format, as long as you give appropriate credit to the original author(s) and the source, provide a link to the Creative Commons license, and indicate if changes were made.
In cases where reviewers are anonymous, credit should be given to 'Anonymous Referee' and the source.
The images or other third party material in this Peer Review File are included in the article's Creative Commons license, unless indicated otherwise in a credit line to the material. If material is not included in the article's Creative Commons license and your intended use is not permitted by statutory regulation or exceeds the permitted use, you will need to obtain permission directly from the copyright holder.

To view a copy of this license, visit https://creativecommons.org/licenses/by/4.0/

Manuscript ID: COMMSBIO-25-4928-T

Response to Reviewers for "Actin conformation dynamics precede force generation in cytotoxic T lymphocytes"

Revision summary:

We are very grateful to the reviewers for their helpful comments and suggestions which improved the quality of our manuscript significantly. We have addressed all the concerns raised by the reviewers and revised the paper. In the following, we have addressed the reviewer's comments point by point in blue, where additions and updates in the manuscript are shown here and in quotation marks and are highlighted in the manuscript in YELLOW.

Reviewer 1:

The manuscript titled "Actin conformation dynamics precede force generation in cytotoxic T lymphocytes" by Rochussen AM et al., provides a valuable insight into the principles of force regulation within the CTL's cortex and the CTL-target interface, i.e., immune synapse. The authors performed a series of careful spatio-temporal analyses of utrophin-CH domain-based F-actin conformation probes to decipher the distribution of the compressed (utrnLAM binding) and tensed (utrnWT binding) filamentous actin.

The key findings are as follows:

1. F-actin conformational changes occur prior to the force generation. Thus, within the causality hierarchy, the actin conformation is upstream of the mechanisms that generate force. I think this is the most important and central finding of the presented work. This raises the question of whether there is a potential feedback regulatory mechanism that links actin conformation to the amount of force within the cytoskeleton.

2. Time coordination of the forces within the immune synapse. Specifically, the transition from compressed F-actin towards tensed F-actin is first observed in the form of the transition from utrnLAM dominance to utrnWT binding.

3. The similarities between artificial IS and the interactions between CTL and live cell targets demonstrate the physiological relevance of these systems.

The work demonstrates substantial experimental and analytical soundness, technical advancement in the dual-expression of F-actin conformation probes in primary TCLs, and a tight and comprehensive integration of the TFM, inhibitor treatments, and live imaging.

The minor criticism that authors may choose to address in the form of discussion points is as follows:

Manuscript ID: COMMSBIO-25-4928-T

Comment #1

Lack of a fully established mechanistic causality: While actin conformation changes precede force generation, it remains unclear whether these changes are necessary and sufficient to drive force production, or merely correlate with upstream signaling events.

Response #1

This is an important point that will be addressed in future work and we wish to make clear to readers. As such, we have included the following paragraph to the discussion (l. 348-352):

"While temporal ordering of events and disruption of force production with inhibitors go some way to elucidating the mechanistic causality here—suggesting that actin conformation is upstream of, but not sufficient for, force production—we were unable to determine whether differential actin conformation is itself necessary for force production. Future studies will be required for further mechanistic insight here."

Comment #2

Although Utrn probes are seemingly optimized and shown not to impair CTL function, their competitive binding with endogenous actin-binding proteins may still subtly change cytoskeletal dynamics. A discussion of possible artifacts or validation with orthogonal probes (e.g., LifeAct mutants or new tension sensors) would strengthen confidence in the results.

Response #2

The potential for small artefacts that do not manifest in the cellular assays we used to compare LifeAct and utrnP2A is valid. We have acknowledged this at the end of the discussion (l. 373-381) now:

"While we showed that broad cellular function is unperturbed by expression of utrnP2A relative to LifeAct-eGFP, we cannot exclude the possibility that binding of utrnWT or utrnLAM does not itself impact actin filament conformation or dynamics, as it is known of Lifeact[59]. Perturbation of the system to enable observation is regrettably unavoidable and the expression of any probe binding to actin could have competition effects with other endogenous actin-binding proteins. Here, further structural work and in vitro assays would be required to assess the mechanistic impact of utrophin probes binding to actin."

Comment #3

Over-reliance on pharmacological treatments. Although blebbistatin (and its derivatives), as well as CK666, are considered relatively specific inhibitors, SmifH2 has numerous off-targets and side effects that cannot always be accounted for in the experiment and the resulting data. What suggestions can authors make in their discussion to address these concerns?

Response #3

Off-target effects of drugs are a valid but often unavoidable concern that we share with the reviewer. We had considered alternatives like siRNA or CRISPR/Cas9, but these require two

rounds of nucleofection (once to perturb gene function and again to express plasmids 24h before imaging) which is not feasible. We have explained this now in the text and offered an alternative path for future experiments by leveraging patient-derived primary T cells (l. 324-330). We have also added references to literature supporting the high specificity of CK666 and para-nitroblebbistatin in particular (l. 328-338).

"Although off-target effects can occur with pharmacological interventions, perturbing the cytoskeleton genetically via electroporation-mediated siRNA or CRISPR/Cas9 was not compatible with electroporation-based plasmid expression. Future work might consider using viral transductions or primary T cells derived from patients harbouring mutations in genes implicated in actinopathies, such as WAS [55], as a means to perturb the cytoskeleton genetically without the need for electroporation.

That said, CK666 and pNB in particular have been shown to be highly specific in vitro and in vivo [56,57], and our experiments with them allowed us to decouple actin network architecture from force production."

Comment #4

Can authors also directly explore the contractility state of the F-actin via immunofluorescent staining of the phospho-myosin (Ser19) and confirm that its spatial distribution matches the tensed F-actin? Even if the two pictures do not match, this would be a valuable exploration of the state of the active (under myosin-driven tension) and passive (hydrostatically induced) tension of the F-actin in the CTL's cortex. Note that this experiment is not mandatory, but it would be desirable for many readers and experts in mechanobiology to see.

Response #4

This is a great suggestion and we have added these data in Figure 5d-e. Our results were intriguing. Phospho-myosin seems to correlate with utrnLAM significantly more than with utrnWT. We have summarized these data in the manuscript (l. 284-293) as follows:

"To gain further insight into the contractility state of the utrophin labelled F-actin, we fixed CTLs expressing utrnP2A and stained them for myosin light chain 2 phosphorylated at serine-19 (p-MLC2). We found p-MLC2 present at the rear of the cells, where it is thought to drive contraction of the actin cytoskeleton that propels migrating T cells [54], and where both utrn probes bind to F-actin similarly (Figure 5d). However, we also found considerable p-MLC2 localisation at the leading edge of migration CTLs, and thus it was colocalised significantly more with utrnLAM than utrnWT across the cells (Figure 5d, e). These data present observational evidence that non-muscle myosin II may be recruited to or involved in the establishment of distinct actin conformations across the cytoskeleton."

Given that these data are only correlative in nature, we do not wish to overclaim at this point. We have added the following to the discussion (l. 307-309 and l. 322-324) to indicate to the reader that further work will be needed to elucidate the role of myosin here:

"Finally, we found that p-MLC2 was spatially coincident with this force-associated utrnLAM-bound F-actin—implying a role for this actin motor protein in establishing or being recruited to distinct actin conformations within the cell."

"In particular, the potential for a spatially coordinated role of non-muscle myosin II in being recruited to or generating either compressed or tensed F-actin will be an exciting future direction."

Reviewer 2:

In light of the growing recognition of the importance of physical forces in T cell function, the authors report that cytotoxic T lymphocytes (CTLs) employ utrophin-based actin conformation probes to visualize F-actin structures and spatial distribution, thereby uncovering a correlation between actin conformation and force generation.

The confocal and TFM imaging data, including both still images and time-lapse videos, are of high quality and effectively support the authors' claims. The use of a P2A sequence to ensure equimolar expression of the two probe variants is technically sound, and the TFM gel system is well-designed, with consistent stiffness closely matching that of cancerous antigen-presenting cells. These methodological strengths add credibility to the conclusions drawn from the experimental results. However, further validation would be necessary before this system can serve as a robust ratiometric reporter of actin filament conformation in live cells, particularly as a potential alternative to widely used probes such as LifeAct.

Comment #1

The authors state that utrnWT preferentially binds to structures such as filopodia, while utrnLAM is enriched in lamellipodia. However, direct evidence demonstrating the selective binding of utrnWT and utrnLAM to filopodia and lamellipodia, respectively, is lacking. If feasible, in vitro reconstitution of branched versus linear F-actin could be performed to validate whether utrnWT and utrnLAM indeed preferentially associate with these distinct actin architectures.

Response #1

This is an important point to clarify. We actually do not find that the actin conformation probes always preferentially bind to filopodia or lamellipodia respectively, but find that probe localization is independent of actin network structure. For example, while it is true that utrnLAM binds strongly to F-actin in lamellipodia for control cells, when we inhibit lamellipodial formation with CK666, we still find enhanced utrnLAM binding at the leading edge of the cell, despite there being no lamellipodia there.

We see the need to clarify our results here, and thank the reviewer for raising this. We have appended the results paragraph (l. 192-195) in question with the following:

"Thus, biased localisation of F-actin conformation is regulated upstream or independently of actin-network structure (e.g. utrnLAM enrichment at the leading edge of migrating cells regardless of lamellipodial presence)."

In vitro reconstitution experiments would be a very insightful extension to this study. We mention this in the discussion but cannot feasibly perform these experiments for this study.

Comment #2

Additionally, while the authors show that regions of dominant utrnWT or utrnLAM localization persist upon treatment with Arp2/3 and formin inhibitors, further clarity could be gained by extending the pharmacological analysis to a broader range of actin-disrupting agents. For instance, treatment with Latrunculin A, which depolymerizes F-actin, or jasplakinolide, which promotes actin polymerization and stabilization, could reveal whether differential probe localization is maintained under a broader range of cytoskeletal perturbations...

Response #2

Since we still observe biased probe localization with our inhibitor treated cells, the suggestion of expanding the repertoire of perturbations to include more drastic cytoskeletal impairments is a fantastic one. We have treated cells with the suggested inhibitors and found that these inhibitors do indeed disrupt biased probe localization across the cell (Figure 5b, c). We have added the following to the results section (l. 270-282):

"Next, we treated CTLs with the actin depolymerization agent latrunculin A and the actin polymerizing and stabilizing drug jasplakinolide. In both instances, we found that the biased probe localisation along the front-rear axis of migrating cells was abolished (Figure 5b, c). As latrunculin A treatment leads to actin depolymerisation, the loss of probe localization is not surprising. Treatment with jasplakinolide on the other hand is known to stabilize actin states—"trapping" the actin conformation in a "young", phosphate bound state and possibly affecting interactions with actin-binding partners. The loss of preferential binding shows that the utrophin probes are binding the actin conformation stabilized by jasplakinolide similarly, with jasplakinolide disrupting the actin confirmation distribution found in untreated cells These findings imply an active process, potentially driven by motor protein-derived tensions, by which the cells establish distinct actin conformations in different areas of the cytoskeleton."

Comment #3

It would also be informative to compare probe localization patterns in drug-treated cells with those in the basal state (untreated and non-migratory conditions), to determine whether the observed differential localization is a specific response to cytoskeletal perturbation rather than a general feature of non-motile cells.

Response #3

This is an excellent suggestion which we thank the reviewer for. An indication of whether the utrn probes are biased in their cellular distribution for a non-migrating cell comes from our artificial synapse experiments, where we imaged cells coming into contact with an anti-CD3ε-coated surface. The probe localisation was already biased at the point of contact (with utrnLAM dominating) (Figure 4). But these images do not reveal the entire cellular distribution. To address this question, we performed imaging of cells in solution far above the glass surface. We found that

Manuscript ID: COMMSBIO-25-4928-T

CTLs express biased probe localisation even without any tactile input of any active migration (Figure 5b, c). We have added the following to the results section (l. 266-270):

"Intrigued by the fact that CTLs exhibit biased utrn probe localisation before coming into contact with both traction force gels and cancerous targets, we sought additional ways to disrupt this bias. By imaging cells floating in solution above the glass surface, we confirmed that CTLs establish polarised actin conformations independently of tactile input (Figure 5b, c)."

Additional changes

We have added to the caption of Figure 5 to reflect our new data there and have added experimental details relating to these new experiments in the Methods section "Immunocytochemistry" (l. 534-544) and lines 581-589 and "Statistics and Reproducibility" (l. 658-662) and updated Data and Code Availability Section (l.689-699). We also updated the authorship section to reflect the new experiments (l.672-674).

We also updated author affiliations.

The new Figure 5 with caption is below:

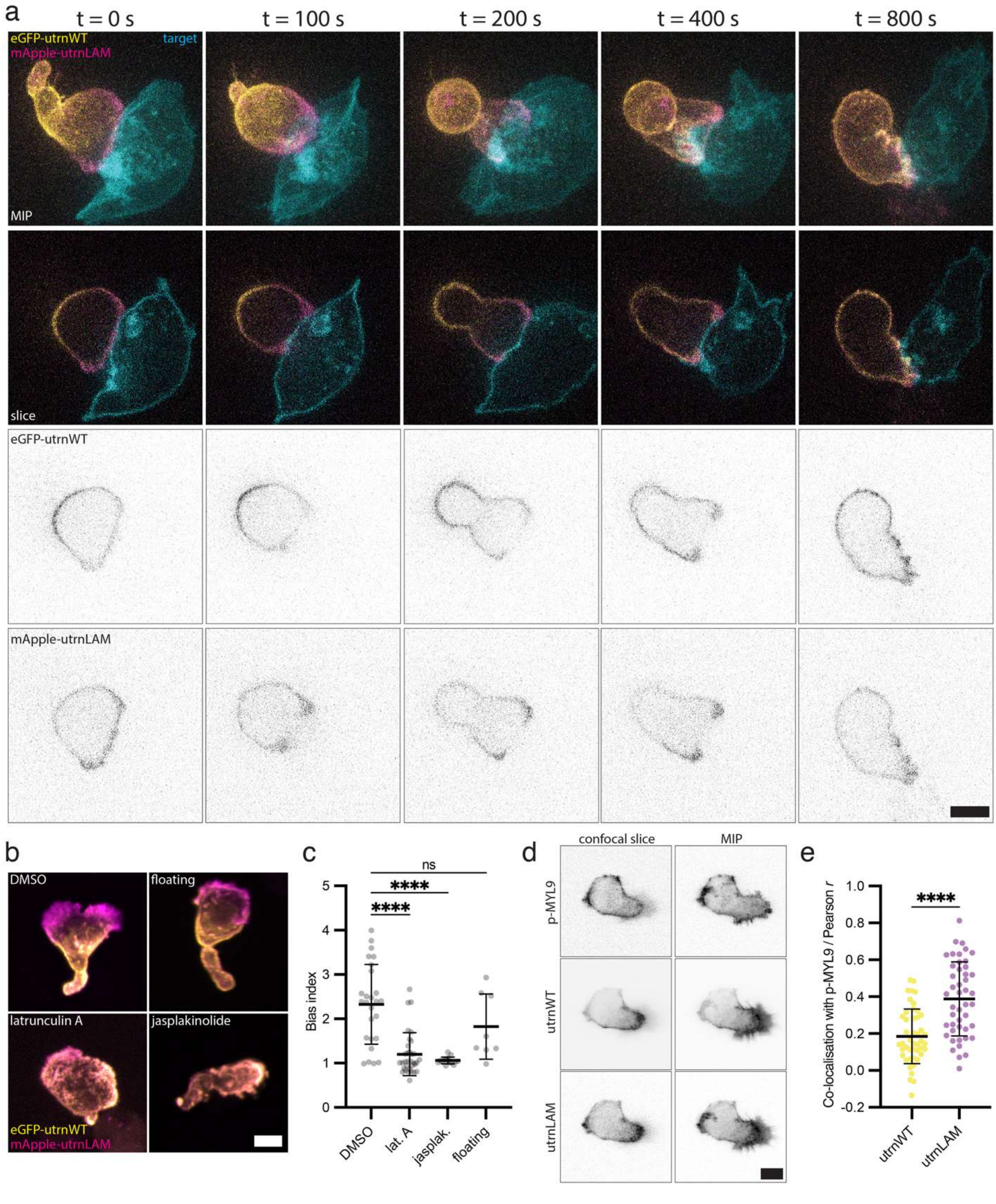

Figure 5 – Polarised actin conformation is dynamic at the cell-cell immune synapse and is associated with phospho-myosin. a: Timelapse spinning disc confocal microscopy of a representative primary OTI CTL, expressing utrnP2A (in merged images: eGFP-utrnWT in yellow, mApple-utrnLAM in magenta) forming an immune synapse with an EL4 target expressing membrane-targeted Electra2 (cyan) that had been pulsed with OVA257-264 peptide. The first

timepoint shown is the point of initial contact between CTL and target. Maximum intensity projections are shown in the top row, and single confocal slices in the other three rows. Representative of 15 cells from across three independent experiments. b-c: Polarised F-actin conformation is present without tactile input but is abolished by latrunculin A and jasplakinolide. b: Composite maximum intensity projections of representative cells expressing utrnP2A as above either treated with DMSO (1:1000), latrunculin A (1 μM), or jasplakinolide (1 μM), or untreated and floating in solution. c: Ratio of normalised probe intensities (LAM/WT) in the front / rear of the cell ("bias index") across conditions in b. Conditions were compared via ordinary one-way ANOVA with Dunnett's multiple comparisons test: DMSO (mean = 2.326, n = 26) versus latrunculin A (mean = 1.202, n = 33) $p < 0.0001$; DMSO versus jasplakinolide (mean = 1.060, n = 16) $p < 0.0001$; DMSO versus floating (mean = 1.825, n = 8) $p = 0.1420$. d-e: Phospho-myosin colocalises with utrnLAM more than utrnWT. d: Single channel images of phospho-MYL9 (ser19) (AF647), eGFP-utrnWT, or mApple-utrnLAM. A single z-slice (in focus with the glass surface) and MIP are shown. e: Colocalisation between p-MYL9 and utrnWT or utrnLAM, determined via Pearson correlation coefficient. Conditions were compared via paired t-test (n = 46): utrnWT (mean = 0.1848) versus utrnLAM (mean = 0.3878) $p < 0.0001$. Scale bar represents 5 μm. Data points represent individual cells pooled from three biologically independent replicates. Error bars represent means and standard deviations.

We believe that the manuscript has benefited from the careful analysis of the reviewers. We appreciate your and their efforts and are pleased to submit this revised version.

With best regards,

Anna Lippert

Manuscript ID: COMMSBIO-25-4928-A

Response to Reviewers for "Actin conformation dynamics precede force generation in cytotoxic T lymphocytes"

Revision summary:

We are highly thankful to the reviewers for their helpful comments and suggestions which improved the quality of our manuscript significantly. We have addressed all the concerns raised by the reviewers and revised the paper. In the following, we have addressed the reviewer's comments point by point **in bold**, where additions and updates in the manuscript are shown here and in blue and are highlighted in manuscript in YELLOW.

Reviewer 1:

The revised manuscript, titled "Actin conformation dynamics precede force generation in cytotoxic T lymphocytes" addresses all raised concerns adequately, reaching the standards of publication. The presented experimental data are now well shaped, the statistical representation and evaluation adequately reflect the strength and rigor of the experimental results, and also support the hypothesis suggested by the authors.

1. The work has been strong and impactful to begin with, but my concerns were mostly about the statistical strength. I now find these concerns properly addressed.

2. The methodology is now better described, and the materials used are sufficiently well identified for reproducibility purposes. The added supplementary figure with the detailed visual and verbal description of the experimental procedure is raising the manuscript to the next level of transparency and experimental reproducibility.

3. I find the presented work important to the field, broadening and deepening our understanding of the T cell cortex mechanobiology, and I believe this publication will be a key to both basic science researchers of the T cell motility/mechanobiology and to the clinical researchers who seek to improve the T cell migrational/invasive properties.

4. The claims that the actin conformational changes are the causal factors for T cell cortex contractility states are novel, important, well presented, and timely for the field.

I generally enjoyed the improved manuscript detail and data presentation

Response

We thank the reviewer for their comments which we feel have greatly improved the manuscript.

Manuscript ID: COMMSBIO-25-4928-A

Reviewer 2:

I have carefully reviewed the revised manuscript and the authors' point-by-point response. I am satisfied with the revisions made, as the authors have addressed all of my previous concerns and queries comprehensively. The manuscript has been significantly strengthened, and I have no further comments or suggestions for improvement at this stage.

Response

We thank the reviewer for their comments which we feel have greatly improved the manuscript.

Reviewer 3:

I have carefully reviewed the revised manuscript and the authors' point-by-point response. I am satisfied with the revisions made, as the authors have addressed all of my previous concerns and queries comprehensively. The manuscript has been significantly strengthened, and I have no further comments or suggestions for improvement at this stage.

Comment #1

The authors report forces that are orders of magnitude higher than reported in the literature. Primary CTLs typically exert forces in the range of 2-20 nN on substrates of stiffness 1.2-6 kPa (Pathni et al., 2022, PMID: 35371019) or micropillar arrays (Basu et al., 2016, PMID: 26924577; other papers from Huse lab). The authors report forces of >10 uN at the cytotoxic immune synapse, which is extremely high! The raw traction values (<10 Pa), though low, seem reasonable given the lack of ICAM-1 in this experimental setup. However, the total forces seem to be erroneously high. A back of the envelope calculation, assuming that the peak tractions are ~6-10 Pa and the cell area is ~100 um2, yields a total force of ~1-2 nN, which is consistent with findings in the literature. It would be informative to see the displacement maps as well. Even adherent cells which exert peak tractions that are 10x that of CTLs have total forces in the 10-100 nN range! The authors should revisit their analysis and revise their conclusions and discussion accordingly as such large discrepancies do not inspire confidence in their main results.

Response #1

We thank the reviewer for raising this point. Having revisited the analysis of the total force we indeed found that the force values were artificially inflated through mapping the force field onto the fluorescent channel, thereby summing the pixels inside the image area, adding the same force values multiple times, rather than summing the force magnitude across the area based on the PIV created force field. We have updated the graph accordingly and arrived at similar values to the reviewers back-of-envelope calculations (peak force is 1-1.5 nN); it did not affect any drawn conclusions.. We have also now removed the discussion paragraph which explored potential reasons for our erroneously high force calculations.

Comment #2

What was the thickness of the hydrogels used? A simple back-of-the-envelope calculation results in a gel thickness of ~12 um when using 3 uL of the gel polymerization of the mix with an 18 mm *coverslip. As per previous literature, this may result in a gel that is too thin and may allow the cells to feel the stiffness of the underlying glass substrate. The authors should comment on the reason this gel thickness was selected, and how this might influence (i) cell behavior, (ii) AFM measurements of gel stiffness, and (iii) any intensity-based ratiometric calculations made through the hydrogel.*

Response #2

We thank the reviewer for their thorough reading. Indeed, this has been a mistake in the method section. The size of top coverslip used was 12 mm across, not 18 mm. The imaging dishes used (Mattek, #P35G-1.5-14-C) have a circular coverslip with a diameter of 14 mm, making an 18 mm top coverslip impossible. We apologize for this error. We had optimized the volume of hydrogel mixture such that the thickness would be large enough to prevent the glass stiffness from being transmitted to the cells. Per our calculations, our gels were theoretically 26.5 µm thick after polymerization. However, hydrogels are known to swell when fully hydrated, often more than doubling in thickness. To measure this, we empirically determined our gel thickness via microscope stage adjustment and have presented these data in a revised supplementary figure 2 (panel d). The average gel thickness was 42.3+/-8.2 µm. Thus, we are confident that our gels were sufficiently thick to allow AFM measurements as performed and to prevent the cells from sensing the stiffness of the glass underneath.

Comment #3

An important paper to cite is Caillier et al., 2024 (PMID: 38889096), where TFM is used to measure forces for T helper cells migrating on PA gels, and these forces are correlated with the localization of focal adhesion proteins such as talin, vinculin and paxillin.

Response #3

We thank the reviewer for bringing this work to our attention and have referred to it in our updated discussion as follows:

"Migration of CD4+ T cells in a confined in vitro 3D environment has recently been shown to be surprisingly mesenchymal in nature, with focal adhesions being required to pull and push T cells forward [Caillier et al ref]. It would be interesting to see how utrophin probe-based actin conformation is distributed in this confined migration model and, ultimately, in vivo."

Comment #4

Can the authors comment on how the respective localization of utrnWT- and utrnLAM-bound F-actin structures would compare to the conventional "bullseye" understanding of the distribution of branched and bundled actin structures at the T cell immune synapse and during T cell migration? utrnLAM-bound F-actin localizes to the branched actin-rich lamellipodia. utrnWT-bound F-actin is present in an annular region of the IS typically associated with actomyosin arcs, but also to actin foci towards the center of the IS which are generated by Arp2/3. Given that localization of these F-actin conformation-specific constructs is upstream of Arp2/3 or formin

activity, how does actin conformation affect the recruitment of these actin-binding proteins? Can the authors comment on these points in the discussion?

Response #4

This is a very interesting point. One of our main findings presented here is that the actin conformation, as you point out, is upstream of Arp2/3, formin, or non-muscle myosin IIa activity (Figure 3). It is a salient hypothesis that these conformations might then influence actin-binding protein recruitment—potentially including NM myosin IIa within the annular region of the immune synapse. In our revised manuscript, we included phospho-myosin staining of migrating T cells and found that it was utrnLAM that correlated with p-MLC2 recruitment. A further exploration of the spatiotemporal relationship between actin conformation and actinomyosin arcs at the synapse would be a very interesting direction for a future study. We have added the following to our discussion:

"In particular, the potential for a spatially coordinated role of actin conformation in the recruitment of non-muscle myosin II in will be an exciting future direction, particularly with respect to actinomyosin arcs at the immune synapse which are known to be formin-dependent [Murugesan et al, JCB, 2016 ref]."

Comment #5

Figure 1: Why was LifeAct used for comparisons for CTL function? When assessing whether expression of an actin probe impacts CTL function, a more appropriate control would be to use cells transfected with a construct that expresses just one or both fluorophores of interest, and additionally, untransfected cells.

Response #5

This is an important point that had been raised by another reviewer too. We had addressed this in our first round of revisions in the discussion as follows:

"While we showed that broad cellular function is unperturbed by expression of utrnP2A relative to LifeAct-eGFP, we cannot exclude the possibility that binding of utrnWT or utrnLAM does not itself impact actin filament conformation or dynamics, as it is known of LifeAct [ref 60]. Perturbation of the system to enable observation is regrettably unavoidable and the expression of any probe binding to actin could have competition effects with other endogenous actin-binding proteins. Here, further structural work and in vitro assays would be required to assess the mechanistic impact of utrophin probes binding to actin."

Untransfected cells do indeed perform better than transfected cells at degranulation and killing, although the degree to which electroporation versus exogenous protein expression versus actin-binding protein over-expression contributes to this difference is not clear (all three likely contribute to some extent). We stand by our decision to use LifeAct as the gold standard benchmark for actin visualization in live cells and hope that the reviewer finds our honest reflection of this in the discussion to be satisfactory.

Comment #6

Figure 2: As presented, the figure just shows an anecdotal example and therefore does not merit a figure by itself. The authors should either present some quantification (potentially of differences in tractions between front and rear of the cell or merge the figure with Figure 3).

Response #6

We have decided against merging with Figure 3 as there is not enough space. Figure 2 establishes how the TFM data is generated and shows a representative cell that is not treated with any drugs. We believe it is useful for readers and contextualizes the subsequent figures. To add to this, we have created a schematic diagram (Figure 2a) to aid understanding of our analysis approach for the broader readership of Communications Biology.

Comment #7

Figure 3: What was the DMSO concentration used for these experiments? How is the quantity (maximum force in the frame) calculated in Fig. 3b (and 3d)? Traction stresses have units of Pa (or kPa) while force has units of Newtons. Is the quantity presented the peak force (which is traction stress x cell area?). Also, the peak values of tractions are in the range of <10 Pa. So how is the value in kPa obtained? The quantity "Mean traction force maximum" in 3d is not defined. Figure 3d should include a legend indicating the conditions corresponding to the different colored symbols for readability. Figure 3e & 4e: The 2D density plots are quite tricky to read and not straightforward to interpret. The authors should explore alternative visualizations that may be easier to interpret. Perhaps calculating a single value of median traction per region (utrnLAM/utrnWT/neutral) per cell, and then comparing these in a violin/swarm chart or paired dot plot?

Response #7

DMSO was used at a 1:1000 dilution (matching the concentration of solvent across our inhibitors). We have added this detail to the methods section.

For figure 3b, we have amended our confusing axis labels. The measurement here is traction force in Pa. Descriptions in the caption reflect PA, but the axes were incorrectly labelled kPa. This has been fixed. This value is the highest traction across all pixels in a particular frame. Data points represent frames, therefore.

For figure 3d, the mean values are simply the means taken from 3b and 3c. A legend depicting which treatments correspond to which colours has now been added, which agree will aid readability. We have also clarified how the data points are derived here in the caption:

"Data points are the means from across all data points and all experiments for each of the four drug treatments corresponding to the central horizontal lines in b and c."

Regarding alternative visualisations, we believe that the additional plots shown in supplementary figure 3b and c satisfy this suggestion. We found 2D density plots to be the most information-rich way of presenting the data and therefore the most transparent way to present the data. We hope that the reviewer is satisfied with the combination of these plots and the aforementioned supplementary plots.

Comment #8

Figure 4:

• *What is the explanation for the utm-WT localization being asymmetric, while the synapse is in general symmetric?*

• *What is the explanation for force being correlated with utrn-LAM localization instead of with utrn-WT? Is this only true for the cell shown or representative of the population? The example in Figure 4a shows that WT is highly enriched in an area of high traction, but this is not apparent in the contour plot. The authors should carry out a correlation or cross-correlation analysis to support this point.*

• *It is remarkable that the pixel-wise traction magnitudes of cells with inhibitors is ~1 Pa, which is essentially background levels (including in their own figure). Multiple studies have shown that blebbistatin does not abolish traction forces (Hui et al. MBoC 2014, Basu et al. Cell 2016) – so the finding that blebbistatin entirely abolishes traction forces is puzzling. The authors should comment on this. The authors should include a supplementary figure showing traction forces and utm-WT and utm-LAM accumulation (similar to Fig 4a) for the inhibitors.*

• *While Figure 4c clearly demonstrates the temporal offset between LAM accumulation and subsequent WT accumulation, it is not clear whether this is a general effect across the population. A temporal cross-correlation analysis for such intensity/traction curves would be necessary to make general conclusions (e.g. see de Jesus et al., Fig. 1-2 for a similar point).*

Response #8

1) We believe that the asymmetric utrnWT distribution is simply biological stochasticity. The synapse is not perfectly circular either, which may be related. The probe localization is very dynamic and one image is only a snapshot of the current actin conformation state while force exertion is seemingly relying on motor protein recruitment, which may well be more symmetrical, but we have not imaged in this experiment.

2) There is some overlap between utrnWT-bound F-actin and high force production in the bottom right of the immune synapse shown, as correctly pointed out by the reviewer. However, there is some density with high traction magnitude in the WT-dominant areas shown in Figure 4f too, which reflects the data shown in Fig 4a. We do not consider these data to be contradictory in the way the reviewer suggests. It is nonetheless true that most force generation is in areas of the synapse that are dominated by utrnLAM-bound actin.

3) While the reviewer rightly points out that some work has shown the reduction but not the complete loss of traction force under Blebbistatin treatment, the loss of traction force under Blebbistatin treatment agrees well with findings of others like the Krummel lab (Nat. Immunology, 2004) where Blebbistatin treatment has been shown to result in complete loss of migration in T cells. Also the work by Basu et al Cell 2016 mentioned by the reviewer in his comment also confirms that traction force under "Blebbistatin treatment dramatically reduced force exertion during both the "spreading" and "squeezing" phases of the response" (Fig. 3G). Even the other work cited by the reviewer (Hui et al) shows a reduced force upon Blebbistatin treatment. However, it should be noted that there are a number of differences between the experiments done by Hui et al., Basu et al., and us that adequately explain the discrepancies that the reviewer highlights.

To summarize the main methodologies of each paper: Hui et al used Jurkat T cells, allowed the cells to spread on 1-2 kPa anti-CD3-coated TFM gels for 15 min, and then added 50 μM

blebbistatin and began imaging right away. Basu et al imaged OTI CTLs on PDMS micropillars coated with both H2-Kb-OVA and ICAM1. They imaged with a 488nm laser every 15 seconds. The timings of their inhibitor treatment (also 50 μM blebbistatin) with respect to imaging is unclear.

The first difference is the drug. Both papers used blebbistatin, which is readily degraded upon exposure to light. Basu et al. additionally imaged with a 488 nm laser, which exacerbates this degradation during the experiment. Hui et al. use a filter to block blue-green light, but is it possible that their blebbistatin solutions were slightly degraded from handling in the lab in the light. We use paranitroblebbistatin, which is a much more photostable drug, and so our active concentration is likely close to the stated concentration (20 μM). A less degraded myosin inhibitor is likely to result in a greater reduction in traction force.

Secondly, Hui et al (and probably also Basu et al) treat the cells shortly before imaging. We treated cells for a full 30 min at 37 degrees C to enable maximal inhibition prior to beginning our imaging. This is because we are mainly interested in the initial actin conformation dynamics upon synapse formation—not the late-stage forces after synapses have already been established. For our synapse imaging, we treated with drug in solution prior to dropping the cells onto the imaging dishes such that we would be able to image the initial point of contact with the gels. Hui et al allow for synapses to form for 15 min before drug treatment. This is why they are able to measure moderate forces with blebbistatin treatment—their T cells had already begun forming immune synapses with the gels. Pre-treating cells before synapse formation, as we did, is expected to result in lower traction forces.

Further, Hui et al use Jurkat T cells—a CD4+ human T cell line—while we used primary mouse T cells. These two cell types may be differently sensitive to drug treatment, with primary mouse cells likely more sensitive. Basu et al, like us, use OTI CTLs, but they use a much more potent gel functionalization strategy. While we used anti-CD3ε, Basu et al used cognate peptide MHC (which has a higher affinity than anti-CD3ε), in addition to ICAM1, which allows for even greater adhesion to traction gels. PDMS micropillars also measure lateral deformation more sensitively compared to our TFM gels.

Despite these differences, which favour higher forces in drug-treated cells in their experiments, they still report a reduction in synaptic force from blebbistatin. Basu et al report “Blebbistatin treatment dramatically reduced force exertion during both the “spreading” and “squeezing” phases of the response”. Hui et al report that myosin II is less important for maintenance of forces at an established synapse, but that it likely plays a greater role in the early stages of synapse formation (which is what we tested in our experiments). When applying blebbistatin only 5 min after synapse formation, they found that “… blebbistatin-treated cells have a significantly lower stress ratio than DMSO (carrier)-treated cells. This confirms that myosin II is required for the initial phase of force generation but is not involved at later time points”.

Therefore, we believe our data with pNB are entirely consistent with both of these publications. Our experimental setup is subtly but importantly different, and we hope the reviewer agrees that our data offers valuable additions to the established literature and does not in fact contradict it.

4) The dynamic actin conformation pattern at the immune synapse was highly reproducible. We agree with the reviewer that it is important to prove this fact. To address this, we have now calculated Pearson’s correlation coefficients for the two utrn probe channels across all replicates.

Our updated figure 4 now contains two new panels (4c and 4d) which depict the increase in co-localisation between the two probes over time at the synapse. 4c related to the representative cell shown, while 4d shows the same trend but using normalised data across replicates. The trend across all replicates was an increase in co-localisation over time ($r_s = 0.454$, $p < 0.0001$), which reflects the initial dominance of utrnLAM followed by utrnWT recovery at the synapse. We have added the following to the results section to describe these data:

"This dynamic pattern of initial utrnLAM dominance at the synapse followed by utrnWT minutes later was detectable via co-localisation analysis between the two probes over time (fig. 4c). In the cell shown, Pearson's r between the two channels began under 0.2. Four minutes after synapse formation, this correlation had risen to above 0.6, where it remained for the duration of the experiment. This pattern was highly consistent, with the co-localisation between probes at the synapse showing a significantly positive trend over time across replicates ($r_s = 0.454$, $p < 0.0001$) (fig. 4d)."

The methods section has also been updated to reflect this additional analysis:

"For colocalization analysis between utrnWT and utrnLAM at the synapse, a time-dependent colocalization channel was generated in Imaris (version 11.0.1, Oxford Instruments) with dynamic automatic thresholding for each channel and timepoint. Pearson's correlation coefficient for each timepoint was extracted. For analysis across replicates, these r values were z-scored for each movie. Spearman's rank-order correlation was used to test for a trend in colocalization over time."

Comment #9

Figure 5: The authors should present summary statistics to demonstrate that conjugates also show similar conformation dynamics. It is unclear what point the authors are trying to make and whether any effect is statistically robust.

Response #9

We have now quantified the utrn probe dynamics at the cell-cell synapse. We employed two strategies: firstly, simply manually drawing polygonal ROIs on MIPs and measuring the mean intensities of each probe over time. Secondly, we used Imaris to generate a 3D volume of the T cell in close proximity to the target cell (i.e., only the part of the T cell closest to the target), and measured sum intensities of each probe channel within this volume over time. We present these data in the revised figure 5b-d. This includes Spearman rank-order correlation analysis to indicate the trend over time of initial utrnLAM dominance followed by utrnWT recovery after several minutes. The figure caption for fig. 5 and the methods section has been updated.

Comment #10

Line 130: Typo in text, should be "at lamellipodia and filopodial tips". Currently listed the other way round.

Response #10

Thank you for this correction.

Manuscript ID: COMMSBIO-25-4928-A

We believe that the manuscript has benefited from the careful analysis of the reviewers. We appreciate your and their efforts and are pleased to submit this revised version.

With best regards,

Anna Lippert
