# Supplementary material for: Actin conformation dynamics precede force generation in cytotoxic T lymphocytes
Source: Commun Biol. 2026 Sep 25;9:1256. doi: 10.1038/s42003-026-11011-3 (PMC13614940; doi:10.1038/s42003-026-11011-3)
Supplement: Supplementary file 3 [file 42003_2026_11011_MOESM2_ESM.pdf]

Supplementary Information

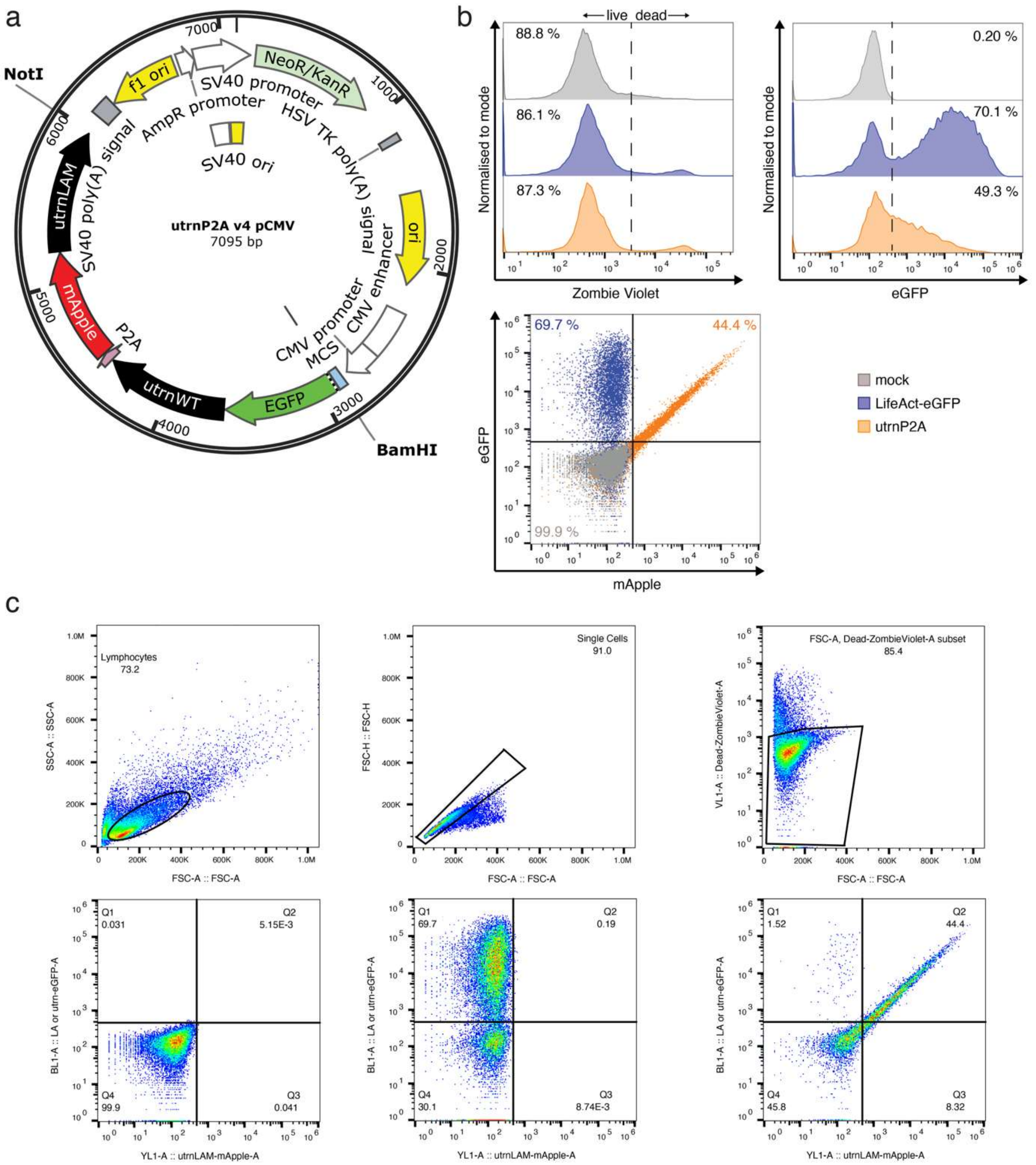

Supplementary Figure 1 - Expressing utrn probes in primary CTLs. a: Plasmid map for utrnP2A construct. Key features are labelled, including restriction sites used for subcloning (BamHI and NotI). **b:** Flow cytometric analysis of CTLs 24 hours after nucleofection with nothing (mock, grey), LifeAct-eGFP (blue), or utrnP2A construct (orange). Histograms depicting viability (top left), eGFP expression (top right), and dot plot comparing eGFP and mApple expression (bottom left). Representative of three

independent experiments. **c**: Gating strategy for degranulation assay (Figure 1d). Expression of plasmids was gated after gating on FSC-A x SSC-A for lymphocytes, FSC-A x FSC-H for singlets, and FSC-A x VL1-A for live cells (using Zombie Violet live/dead stain). LAMP1 (BV711) fluorescence was assessed for cells in the Q1 gate of the BL1-A x YL1-A channel for LifeAct-eGFP-expressing cells and Q2 for utrnP2A-expressing cells. Compensation was not necessary for this panel.

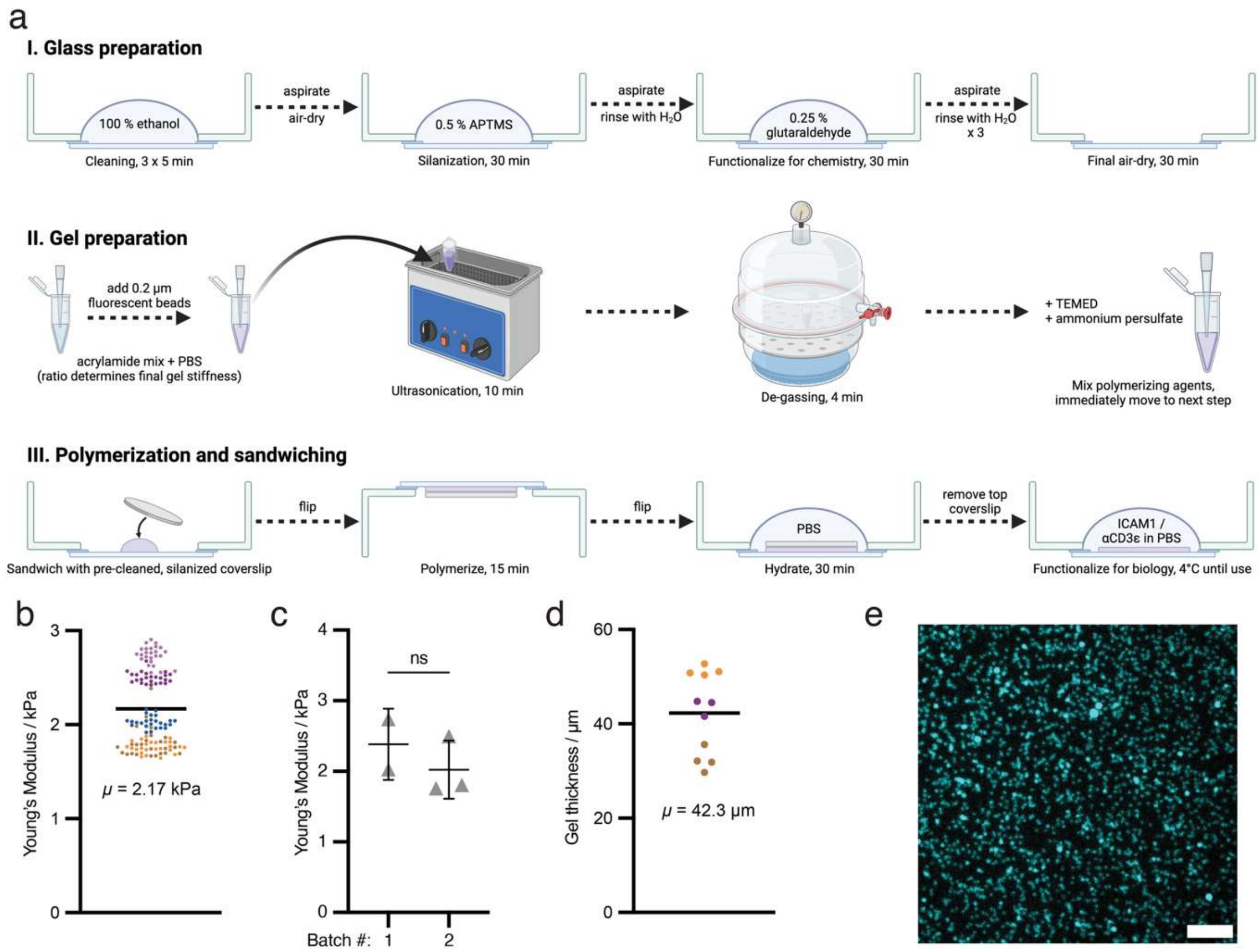

Supplementary Figure 2 - Synthesis and validation of traction force microscopy gels. a: Schematic diagram summarising the protocol for synthesis of traction force microscopy gels. Made in BioRender. **b-c**: Atomic force microscopy measurements of gel stiffness. Representative gels were selected from successive batches for AFM measurements. **b:** Stiffness measurements across five gels (each coloured differently). Twenty-five measurements were made per gel, sampling from the full area of the gel. The population mean is shown. **c:** Inter-batch consistency. Data points are means of 25 measurements per gel. Bars depict mean and standard deviation. There was no significant difference between batches (two-tailed t test, p = 0.44). **d:** Empirical measurement of gel thickness for three representative gels, each measured at 3-4 areas across the gel surface. The population mean is shown. **e:** Representative field of view of a TFM gel, imaged with a spinning disc confocal microscopy. Even distribution of dark red FluoSpheres™ (cyan) embedded beneath the surface can be seen. Scale bar represents 5 µm

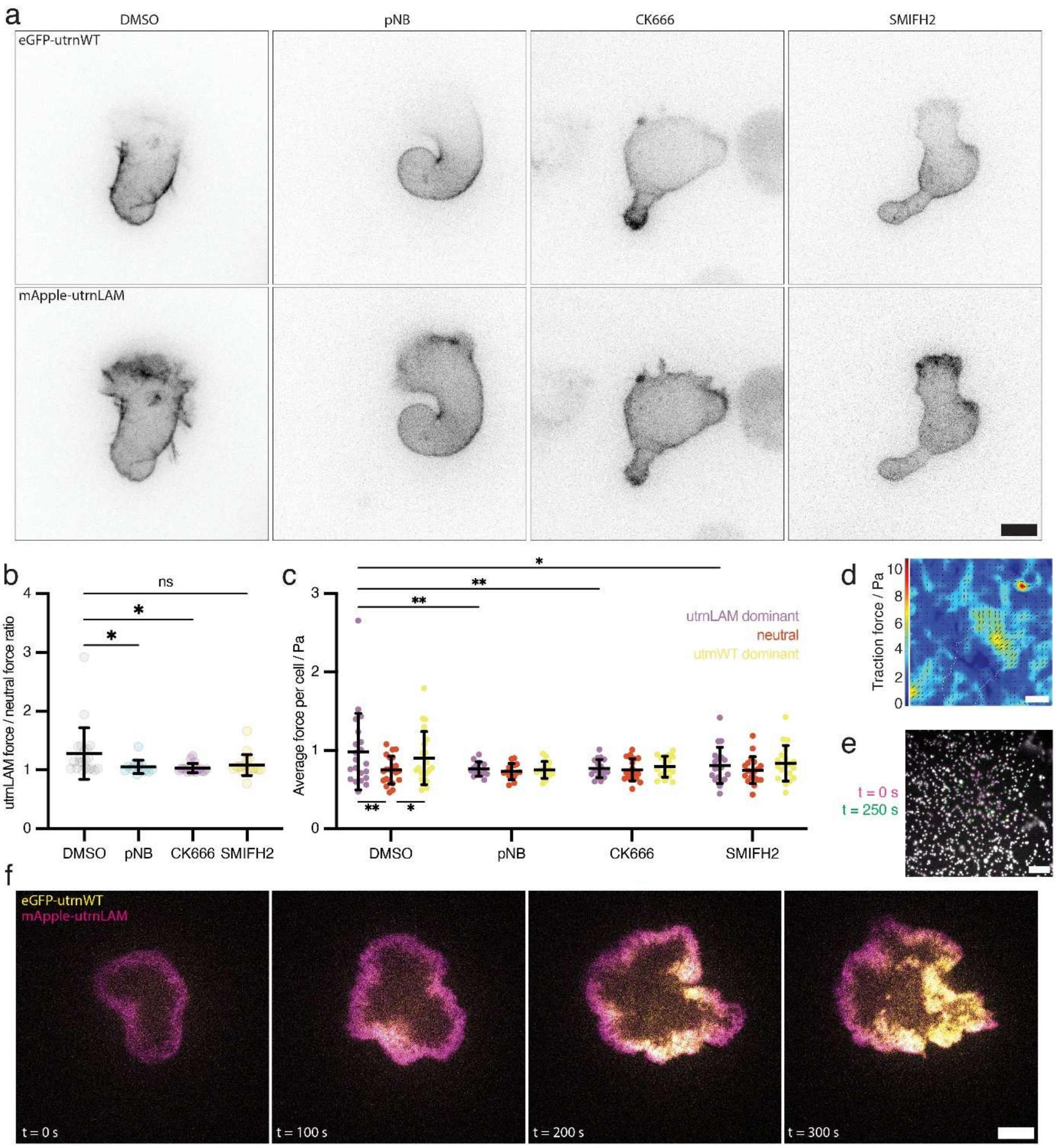

Supplementary Figure 3 – Abrogation of force production and actin network structures does not affect biased probe localisation. a: OTI CTLs expressing utrnP2A crawling on ICAM-coated TFM gels treated with the indicated drugs. Separated channel images in inverted greyscale relating to figure 3a. **b**: Ratio of average traction force value per movie between utrnLAM dominant and neutral cell areas between different drug treatments. Data points represent time lapse imaging series of individual cells. Bars represent mean and standard deviations. Conditions were compared via one-way ANOVA with Dunnett's multiple comparisons test: DMSO (mean = 1.280) vs pNB (mean = 1.053) p = 0.0409; DMSO vs CK666 (mean = 1.031) p = 0.0146; DMSO vs SMIFH2 (mean = 1.081) p = 0.0549. **c**: Comparison of average

traction force per movie per segmented cell area between different drug treatments. Cells were segmented into three areas based on ratio of utrn probe intensity. Average force production in each area across time lapse imaging was calculated. Bars represent mean and standard deviations. Conditions and cell areas were compared via two-way ANOVA and Šídák's multiple comparisons test: utrnLAM dominant vs neutral DMSO, p = 0.0052; utrnLAM dominant DMSO vs pNB, p = 0.0466; utrnLAM dominant DMSO vs CK666, p = 0.0374; utrnLAM dominant DMSO vs SMIFH2, p = 0.1239. **d**: Representative traction force field with directional arrows for a DMSO-treated OTI CTL crawling on ICAM-1 coated TFM gel relating to Figure 3a. Scale bar represents 5 μm. Traction force is colour coded in Pa. Cell outline is shown in dotted white. **e**: Overlaid images of traction force beads from frame 1 (magenta) and frame 25 (green) relative to initial contact of the cell with the gel, relating to a. Where no bead movement has occurred, co-localisation results in white beads. Where there has been movement, the colours are seen separately. **f:** An OTI CTL expressing utrnP2A (eGFP-utrnWT in yellow, mApple-utrnLAM in magenta) forming an immune synapse with an anti-CD3ε-coated TFM gel. Merged channel image relating to Figure 4a.

Supplementary Movies

Supplementary Movie 1 - Actin conformation probes exhibit biased localisation in migrating CTLs. Time-lapse spinning disc confocal microscopy of an OTI CTL crawling on ICAM1-coated glass. The cell is expressing eGFP-utrnWT (yellow) and mApple-utrnLAM (magenta) from the utrnP2A construct. Maximum intensity projections shown. Scale bar represents 5 μm. Time in mm:ss format. Representative of at least 30 cells from at least five independent experiments.

Supplementary Movie 2-5 - Traction force microscopy of migrating CTLs expressing actin conformation probes. Time-lapse spinning disc confocal microscopy of an OTI CTL, expressing utrn probes (utrnWT, yellow; utrnLAM, magenta) crawling on an ICAM-coated TFM gel (AF647 microsheres, cyan) (E = 2.17 kPa). Representative maximum intensity projections shown. Scale bar represents 5 μm. Time is given in seconds. Cells were treated with DMSO (Movie 2) (N = 3, n = 21),

paranitroblebbistatin (Movie 3) (N = 3, n = 14), CK666 (Movie 4) (N = 3, n = 17), or SMIFH2 (Movie 5) (N = 3, n = 19).

Supplementary Movie 6 – Dynamic changes to actin conformation at the cytotoxic immune synapse. Time-lapse spinning disc confocal microscopy of an OTI CTL, expressing utrn probes (utrnWT, yellow; utrnLAM, magenta) forming a synapse with an anti-CD3ε-coated TFM gel (AF647 microsheres, cyan) (E = 2.17 kPa). Cell channels (left) and drift-corrected bead channel (right) are separated to better visualize bead movement. Single confocal slice in plane with the gel surface was acquired. Scale bar represents 5 µm. Time given in mm:ss format. N = 3, n = 5.

Supplementary Movie 7 – Traction force at the cytotoxic immune synapse. Traction force reconstruction relating to supplementary movie 6. Each frame is 10 seconds apart, and the image is 35 µm wide. Colour-coded traction is in units of Pascals.

Supplementary Movie 8-9 – Dynamic changes to actin conformation at the physiological cytotoxic immune synapse. Timelapse spinning disc confocal microscopy of a representative primary OTI CTL, expressing utrnP2A (eGFP-utrnWT in yellow, mApple-utrnLAM in magenta) forming an immune synapse with an EL4 target expressing membrane-targeted Electra2 (cyan) that had been pulsed with $OVA_{257\text{-}264}$ peptide. Maximum intensity projections (Movie 8) and a single confocal slice through the centre of the synapse (Movie 9) are shown. Scale bar represents 5 µm. Representative of 15 cells from across three independent experiments.
