# Supplementary material for: Actin conformation dynamics precede force generation in cytotoxic T lymphocytes
Source: Commun Biol. 2026 Sep 25;9:1256. doi: 10.1038/s42003-026-11011-3 (PMC13614940; doi:10.1038/s42003-026-11011-3)
Supplement: Supplementary file 4 [file 42003_2026_11011_MOESM3_ESM.pdf]

Description of Additional Supplementary Files:

File: Supplementary Data

Description: Tabular data for all graphs

File: Supplementary Movie 1

Description: Actin conformation probes exhibit biased localisation in migrating CTLs. Time-lapse spinning disc confocal microscopy of an OTI CTL crawling on ICAM1-coated glass. The cell is expressing eGFP-utrnWT (yellow) and mApple-utrnLAM (magenta) from the utrnP2A construct. Maximum intensity projections shown. Scale bar represents 5 μm. Time in mm:ss format. Representative of at least 30 cells from at least five independent experiments.

File: Supplementary Movie 2

Description: Traction force microscopy of migrating CTLs expressing actin conformation probes treated with DMSO. Time-lapse spinning disc confocal microscopy of an OTI CTL, expressing utrn probes (utrnWT, yellow; utrnLAM, magenta) crawling on an ICAM-coated TFM gel (AF647 microsheres, cyan) (E = 2.17 kPa). Representative maximum intensity projections shown. Scale bar represents 5 μm. Time is given in seconds. Cells were treated with DMSO (N = 3, n = 21).

File: Supplementary Movie 3

Description: Traction force microscopy of migrating CTLs expressing actin conformation probes treated with pNB. Time-lapse spinning disc confocal microscopy of an OTI CTL, expressing utrn probes (utrnWT, yellow; utrnLAM, magenta) crawling on an ICAM-coated TFM gel (AF647 microsheres, cyan) (E = 2.17 kPa). Representative maximum intensity projections shown. Scale bar represents 5 μm. Time is given in seconds. Cells were treated with paranitroblebbistatin (N = 3, n = 14).

File: Supplementary Movie 4

Description: Traction force microscopy of migrating CTLs expressing actin conformation probes treated with CK666.Time-lapse spinning disc confocal microscopy of an OTI CTL, expressing utrn

probes (utrnWT, yellow; utrnLAM, magenta) crawling on an ICAM-coated TFM gel (AF647 microsheres, cyan) (E = 2.17 kPa). Representative maximum intensity projections shown. Scale bar represents 5 μm. Time is given in seconds. Cells were treated with CK666 (N = 3, n = 17).

File: Supplementary Movie 5

Description: Traction force microscopy of migrating CTLs expressing actin conformation probes treated with SMIFH2. Time-lapse spinning disc confocal microscopy of an OTI CTL, expressing utrn probes (utrnWT, yellow; utrnLAM, magenta) crawling on an ICAM-coated TFM gel (AF647 microsheres, cyan) (E = 2.17 kPa). Representative maximum intensity projections shown. Scale bar represents 5 μm. Time is given in seconds. Cells were treated with SMIFH2 (N = 3, n = 19).

File: Supplementary Movie 6

Description: Dynamic changes to actin conformation at the cytotoxic immune synapse. Time-lapse spinning disc confocal microscopy of an OTI CTL, expressing utrn probes (utrnWT, yellow; utrnLAM, magenta) forming a synapse with an anti-CD3ε-coated TFM gel (AF647 microsheres, cyan) (E = 2.17 kPa). Cell channels (left) and drift-corrected bead channel (right) are separated to better visualize bead movement. Single confocal slice in plane with the gel surface was acquired. Scale bar represents 5 μm. Time given in mm:ss format. N = 3, n = 5.

File: Supplementary Movie 7

Description: Traction force at the cytotoxic immune synapse. Traction force reconstruction relating to supplementary movie 6. Each frame is 10 seconds apart, and the image is 35 μm wide. Colour-coded traction is in units of Pascals.

File: Supplementary Movie 8

Description: Dynamic changes to actin conformation at the physiological cytotoxic immune synapse. Timelapse spinning disc confocal microscopy of a representative primary OTI CTL, expressing utrnP2A (eGFP-utrnWT in yellow, mApple-utrnLAM in magenta) forming an immune synapse with an EL4 target expressing membrane-targeted Electra2 (cyan) that had been pulsed with OVA257-264 peptide. Maximum intensity projections are shown. Scale bar represents 5 μm. Representative of 15 cells from across three independent experiments.

File: Supplementary Movie 9

Description: Dynamic changes to actin conformation at the physiological cytotoxic immune synapse. Timelapse spinning disc confocal microscopy of a representative primary OTI CTL, expressing utrnP2A (eGFP-utrnWT in yellow, mApple-utrnLAM in magenta) forming an immune synapse with an EL4 target expressing membrane-targeted Electra2 (cyan) that had been pulsed with OVA257-264 peptide. A single confocal slice through the centre of the synapse is shown. Scale bar represents 5 μm. Representative of 15 cells from across three independent experiments.
